# Scanning central carbon metabolism: a HILIC-HR-TOF-MS metabolome method

**DOI:** 10.1007/s11306-026-02434-4

**Published:** 2026-05-13

**Authors:** Victoria Pozo Garcia, Valentina Ferro, Jolene Rier, Sofia Moco

**Affiliations:** Department of Chemistry and Pharmaceutical Sciences, Amsterdam Institute of Molecular and Life Sciences (AIMMS), Vrije Universiteit Amsterdam, Amsterdam, The Netherlands

**Keywords:** LC–MS, Metabolomics, Central carbon metabolism, HILIC

## Abstract

**Introduction:**

Central carbon metabolism (CCM) is the primary metabolic hub of the cell, governing energy production and providing precursors essential for a myriad of biosynthetic pathways. Developing analytical tools that can identify and quantify intermediates of these metabolic reactions is crucial for studying cell metabolism in biomedical and biotechnological applications.

**Objective:**

This study proposes a liquid chromatography (LC)-high-resolution (HR) mass spectrometry (MS) method, covering the CCM of mammalian cell systems.

**Methods:**

Cells were extracted using a one-step liquid extraction, recovering the hydrophilic metabolites. A stable isotope dilution approach was employed, utilizing a U-^13^C-yeast internal standard (IS). A LC-HRMS metabolomics method using hydrophilic interaction liquid chromatography (HILIC) coupled to a Zeno-time-of-flight (ZenoTOF) MS was implemented for metabolite semi-quantification.

**Results:**

A total of 82 CCM metabolites is reported, of which 77 were confirmed with authentic standards, and for 63 , linearity ranges were obtained. IS normalization enhanced overall robustness, from sample preparation to metabolite semi-quantification. To study the effects on CCM by 5 chemical inhibitors (2-deoxy-d-glucose, etomoxir, UK-5099, rotenone, and 3-nitropropionic acid), our HILIC-HR-TOF-MS method was used. The approach proved efficient in capturing altered metabolite concentrations, within implicated metabolic reactions, as a consequence of inhibitor exposure.

**Conclusion:**

Our HILIC-HR-TOF-MS metabolome method is efficient in mapping changes in metabolic intermediates of the CCM in mammalian cells. This approach holds potential for analysing a variety of biological samples across a range of applications, from drug development to biomedicine.

**Supplementary Information:**

The online version contains supplementary material available at 10.1007/s11306-026-02434-4.

## Introduction

Central carbon metabolism (CCM) is the primary metabolic hub of the cell, encompassing key pathways such as glycolysis, the pentose phosphate pathway (PPP), the tricarboxylic acid (TCA) cycle, and surrounding reactions. These metabolic pathways catabolize carbon sources, including glucose, to produce energy in the form of ATP or reducing equivalents, which in turn fuel mitochondrial metabolism and oxidative phosphorylation (OXPHOS). Moreover, CCM also comprises essential metabolic intermediates that act as precursors in many anabolic processes, for instance, amino acid and nucleotide biosynthesis, as well as fatty acid oxidation (Berg et al., 2019).

The development of analytical techniques capable of measuring metabolic intermediates of the CCM is thus essential for understanding cellular bioenergetics and homeostasis in various applications, ranging from biotechnology to metabolic health and disease. Analytical techniques, such as nuclear magnetic resonance (NMR) (Moco, 2022; Wishart, 2008), liquid chromatography (LC)-mass spectrometry (MS) (Bajad et al., 2006; Buescher et al., 2010), direct flow injection-MS (Fuhrer et al., 2011), gas chromatography-MS (Eylem et al., 2022), or capillary electrophoresis-MS (Osanai et al., 2014) have been used for this purpose. LC–MS techniques are the most common choice for performing metabolomics analysis of the CCM. This is due to LC–MS’s high sensitivity and broad coverage for analyzing low molecular weight compounds in biological extracts, without the need for sample derivatization (Cajka & Fiehn, 2016; Gorrochategui et al., 2016).

Different LC methods have been employed for the study of CCM intermediates: ion pairing (Buescher et al., 2010), ion exchange (Ngere et al., 2023), reverse phase (RP) (Lu et al., 2006), mixed mode (MMC) (Kozaki, 2025) or hydrophilic interaction liquid chromatography (HILIC) (Chareyron et al., 2020; Pluskal et al., 2010). While initial approaches have relied on C18 RP chromatography (Lu et al., 2006), these resulted in suboptimal conditions, as polar metabolites failed to be appropriately retained in the column (Girel et al., 2023). HILIC has then emerged as an alternative to separate a broad range of hydrophilic cellular metabolites, including amino acids, nucleotides, carboxylic acids, and sugar phosphates, without the need for ion pairing agents (Cubbon et al., 2010; Girel et al., 2023; Lu et al., 2008). HILIC separation is achieved by balancing analyte hydrophilic and weak electrostatic interactions. Typical HILIC gradients consist of organic solvents in water, with acetonitrile being more common than methanol, resulting in narrower peaks due to its lower viscosity (Cubbon et al., 2010). Buffers used in HILIC are often ammonium salts of acetate and formate (5–20 mM), soluble at high organic percentage (Cubbon et al., 2010). In addition to optimal chromatography separations, appropriate sample preparation methods are key in the detection of CCM metabolites, given that some of these analytes are inherently unstable (e.g., redox cofactors, energy metabolites) (Bylda et al., 2014).

Tandem MS instruments, such as triple quadrupoles (QqQ-MS/MS), are often the preferred choice for targeted metabolomic studies, enabling high sensitivity and absolute quantification through selected or multiple reaction monitoring (SRM or MRM) acquisition. For this approach, extensive method development is required, for which the MS parameters of each analyte are optimized to achieve the highest selectivity and sensitivity. However, an alternative strategy is the use of spectrometers allowing for high-resolution MS (HRMS), such as time-of-flight (TOF)-MS or Orbitrap-MS, for a wide coverage of analytes, resulting in a large-scale accurate mass separation of ions (Gorrochategui et al., 2016; Lu et al., 2008). HRMS has proven to be reliable for performing qualitative, as well as quantitative studies, recording HR full scan or MS/MS mode (Grund et al., 2016). As benefits, HR-MS acquisition in full scan using metabolite lists bridges the gap between targeted and untargeted analysis: a “semi-targeted” analysis, providing a more comprehensive overview of the sample composition, that does not require extensive method development (Grund et al., 2016; Lu et al., 2008). Furthermore, this strategy enables the re-interrogation of acquired LC-HRMS data for previously unidentified metabolites, thereby allowing progressive characterization of biological systems through MS-based metabolomics analysis.

With advances in LC-HRMS technology, datasets have become increasingly large and complex (Gorrochategui et al., 2016), emphasizing the need for efficient metabolite identification strategies. Specifically, downstream metabolomics data requires a time-consuming characterization of metabolites’ analytical properties with authentic standards, prior to sample acquisition (Gorrochategui et al., 2016; Patti et al., 2012). Thus, having a reference database can accelerate metabolite identification, even in interlaboratory settings (Gorrochategui et al., 2016).

In this study, an LC-HRMS metabolomics workflow using HILIC and full-scan MS was developed to identify 81 metabolites from the CCM. Seventy-seven metabolites were confirmed using authentic standards, and the limit of detection (LOD) was obtained for 63 metabolites in one or both ion modes. To evaluate method performance, the analytical pipeline reported here was employed to assess metabolic disturbances in the intracellular metabolome of a hepatic cell system challenged with a set of CCM inhibitors. Ultimately, this work aims to serve as a reference point for covering CCM metabolic intermediates in cell studies, with the potential to be expanded and applied to other biological samples and metabolites.

## Materials and methods

### Reagents and chemicals

All solvents used in metabolomics were LC-MS grade (Biosolve), and ultrapure water was obtained from a water purification system (Milli-Q EQ 7000, Merck Life Science). Inhibitors used for cell culture experiments were purchased from Sigma: 2-deoxy-d-glucose (CAS. 154-17-6), etomoxir (CAS. 828934-41-4), UK-5099 (CAS. 56396-35-1), 3-nitropropionic acid (CAS. 504-88-1), and rotenone (CAS. 83-79-4). DMSO (CAS. 67-68-5) was purchased from Merck. Suppliers for all authentic standards used in method development are listed in Table S1.

### Authentic standard compound solutions

Seventy-six authentic standards (CCM metabolites) were weighed and dissolved in water or methanol to achieve stock concentrations ranging from 0.3 to 10 mM, depending on the compound’s solubility (Table S1). The nucleotide phosphates uridine triphosphate (UTP) and cytidine triphosphate (CTP) were heated to obtain diphosphates and monophosphates. Most of the standard compounds were combined into multi-compound solutions, separating the metabolites based on their nominal mass. Standard curves of the multi-compound solutions were prepared in 60% (v/v) acetonitrile/ultrapure water at 9 concentrations, ranging from 0.08 to 20 µM, with a 1:2 dilution series. Standard solutions were analysed by LC-MS.

### Cell culture

Human hepatoma HepaRG cells were obtained from Biopredic International (Saint Grégoire, France) as undifferentiated cells. Cells were cultured in William’s E medium, supplemented with 2 mM GlutaMAX^TM^ (Gibco, cat. 32551020), 1% (v/v) penicillin/streptomycin (Sigma, cat. P4333), 9% (v/v) fetal bovine serum (FBS) (Gibco, cat. 10270106), 50 µM hydrocortisone 21-hemisuccinate (Sigma, CAS. 125-04-2) and 5 µg/mL human insulin (Sigma, CAS. 11061-68-0). Cells were passaged with trypsin (Sigma, cat. T4049) and seeded with a density of 45.000 cells/cm^2^. Forty-eight hours after seeding, cells were differentiated as previously described (Pozo Garcia et al., 2025) by supplementing the cultured media with 1.7% (v/v) DMSO for a week. Media was exchanged every 48 h (72 h over the weekend) and right before inhibitor treatment. Cells used in the experiment were passage 29 and tested 1 day after differentiation. Cells were maintained in an incubator at 37 °C under a humidified atmosphere with 5% CO_2_.

### Cell treatment and sample preparation

HepaRG cells were incubated for 24 h with CCM inhibitors: 10 mM 2-deoxy-d-glucose, 30 µM etomoxir, 10 µM UK-5099, 100 µM 3-nitropropionic acid, and 5 nM rotenone. To utilize appropriate inhibitor concentrations, cell viability was assessed using the resazurin assay (Limonciel et al., 2011) assuring the dosed concentrations stayed below the cytotoxicity level (Fig. S1). Treatment was performed in 6-well plate format (2 mL/well) with a final concentration of 0.1% (v/v) DMSO for all conditions. After 24 h exposure, media was discarded, and cells were washed with 0.9% (m/v) NaCl and immediately quenched in liquid nitrogen. Intracellular contents were extracted by scraping the cells with 1.5 mL 80% (v/v) MeOH/water, with in-house produced U-^13^C yeast extract internal standard (IS) (Mashego et al., 2004). Samples were agitated at 4 °C for 30 min in an Eppendorf Thermomixer R 1.5mL Shaking Heater Block, followed by a centrifugation step (15 min), before overnight evaporation in a vacuum centrifuge (CHRIST). The dry lysates were resuspended in 100 µL 60% (v/v) acetonitrile/water, and centrifuged (5 min) before LC-MS analysis.

### LC-HRMS analysis

Separation was performed using an Agilent 1290 ultra-high performance liquid chromatograph (UHPLC) consisting of a high-pressure pump (G7120A), an autosampler (G7129B), and a column compartment (G7116B) coupled to a SCIEX ZenoTOF 7600 MS system with a heated electrospray ionisation source (Turbo V ion source). The method was adapted from Chareyron et al ( 2020). HILIC was used for the chromatographic separation with a ZIC®-pHILIC column (5 µM, polymeric, 150 × 2.1 mm) and a guard column ZIC®-pHILIC (5 µM, polymeric, 20 × 2.1 mm) at 35 °C. The eluents used: A (water: 10 mM ammonium acetate (NH_4_Ac) with 0.04% (v/v) ammonium hydroxide; pH~9.5–10) and B (100% acetonitrile) were pumped at a flow rate of 0.2 mL/min. A gradient was applied from 90 to 25% B in 21 min, with a total run of 30 min, including washing and re-equilibration (0-0.50 min: 90% B; 0.50-16 min: 25% B; 16-21 min: 25% B; 21.10-30 min: 90% B). The injection volume was 3 µL, and samples were maintained at 10 °C until analysis. MS acquisition was performed in full-scan TOF-MS between 50 and 1000 Da. The source parameters were the following: nebulizer and drying gas pressure at 40 psi, curtain gas and the collisionally activated dissociation (CAD) gas were at 35 and 8 psi, respectively. Source temperature was set at 400 °C. Capillary voltage was 5.5 kV for positive and 4.5 kV for negative ion modes, with a declustering potential of 50 and 80 V, respectively. Collision energy was set at 10 V for both polarities. The accumulation time was 0.1 s. MS calibration was performed before and during the run (every 7–8 samples) using the provider’s calibration solution (SCIEX). For the LC-MS analysis of cell extracts, the sample order was randomized, and quality control samples (QCs) were injected every 6 samples. QCs consisted of a pool of samples under all experimental conditions. Data acquisition was performed in profile mode. Acquisition and data pre-processing were done under the control of SCIEX OS V.3.1.6 software. Specific metabolite features (Table 1) were integrated with SCIEX’s AutoPeak algorithm from LC-MS data, using a targeted list of expected molecular ions, matched for ± 5 ppm.

**Table 1:** LC-HRMS properties of central carbon metabolism intermediates

Pathway	Compound common name	Chemical formula	RT (min)	[H+M]^+^ theoretical	[H−M]^−^theoretical	Mass error (+)	Mass error (−)	IS	Intracellular presence	ACS	LOD ^+^ (μM)	LOD^-^ (μM)
AA	Glutamine	C5H10N2O3	8.93	147.07642	145.06187	0.4	0.3	U-^13^C_Glutamine	X	X	0.08	0.08
AA	Alanine	C3H7NO2	8.66	90.05495	88.04040	2	0.2	U-^13^C_Glutamine	X	X	0.6	10
AA	Arginine	C6H14N4O2	15.19	175.11895	173.10440	0.3	0.3	U-^13^C_Arginine	X	X	0.08	1.25
AA	Aspartate	C4H7NO4	9.32	134.04478	132.03023	0	0.2	U-^13^C_Glutamine	X	X	0.3	0.15
AA	Glutamate	C5H9NO4	9.38	148.06043	146.04588	0.1	0.1	U-^13^C_Glutamine	X	X	0.08	0.08
AA	Glycine	C2H5NO2	9.16	76.03930	74.02475	9.3	0.3	U-^13^C_Glutamine	–	X	5	10
AA	Histidine	C6H9N3O2	8.73	156.07675	154.06220	0.2	0.1	U-^13^C_Glutamine	X	X	0.08	0.08
AA	Isoleucine	C6H13NO2	6.86	132.10191	130.08735	0.1	0.2	U-^13^C_Glutamine	X	X	0.08	0.15
AA	Leucine	C6H13NO2	6.64	132.10191	130.08735	0.1	0.6	–	Adjacent to isoleucine	X	0.08	0.15
AA	Lysine	C6H14N2O2	14.62	147.11280	145.09825	0.3	0.5	U-^13^C_Glutamine	X	X	0.3	2.5
AA	Methionine	C5H11NO2S	7.18	150.05833	148.04377	0	0	U-^13^C_Glutamine	X	X	0.08	0.6
AA	Phenylalanine	C9H11NO2	6.48	166.08626	164.07170	0.1	0	U-^13^C_Glutamine	X	X	0.08	0.15
AA	Proline	C5H9NO2	7.52	116.07061	114.05605	0.5	0.4	U-^13^C_Glutamine	X	X	0.08	0.3
AA	Serine	C3H7NO3	9.27	106.04987	104.03532	0.1	0.2	U-^13^C_Glutamine	X	X	0.15	0.6
AA	Threonine	C4H9NO3	8.65	120.06552	118.05097	0.6	0.1	U-^13^C_Glutamine	X	X	0.08	0.15
AA	Tyrosine	C9H11NO3	7.95	182.08117	180.06662	0	0.1	U-^13^C_Glutamine	X	X	0.08	0.15
AA	Valine	C5H11NO2	6.63	118.08626	116.07170	0.8	0	U-^13^C_Glutamine	X	X	1.25	0.15
AA	Tryptophan	C11H12N2O2	7.12	205.09715	203.08260	0	0.1	U-^13^C_Glutamine	X	X	0.3	0.08
AA	Asparagine	C4H8N2O3	9.05	133.06077	131.04622	0.1	0.7	U-^13^C_Glutamine	X	X	0.08	0.15
AA	Phosphoserine	C3H8NO6P	10.44	186.01620	184.00165	0.3	–	U-^13^C_Glutamine	X	X	–	–
AA	Kynurenine	C10H12N2O3	6.85	209.09207	207.07752	0.1	–	U-^13^C_Glutamine	X	X	–	–
AA	Citrulline	C6H13N3O3	9.36	176.10297	174.08842	0.1	0.1	–	–	X	0.08	0.08
AA	Ornithine	C5H12N2O2	13.51	133.09715	131.08260	2.3		U-^13^C_Glutamine	X	X	–	–
AA	Taurine	C2H7NO3S	8.77	126.02194	124.00739	0.3	0.2	U-^13^C_Glutamine	X	X	0.15	0.08
Glycolysis	Glucose-6-phosphate (G6P)	C6H13O9P	10.53	261.03700	259.02244	0	0.1	U-^13^C_G6P	X	X	0.6	0.08
Glycolysis	Fructose-6-phosphate (F6P)	C6H13O9P	11.25	261.03700	259.02244	0.3	0.1	U-^13^C_G6P	X	X	5	0.08
Glycolysis	Fructose-1,6-bisphosphate (F1,6BP)	C6H14O12P2	12.20	341.00333	338.98877	0	0.2	U-^13^C_G6P	X	X	5	0.15
Glycolysis	Glyceraldehyde-3-phosphate (G3P)	C3H7O6P	9.94	171.00530	168.99075	–	3.2	U-^13^C_G6P	X	X	–	10
Glycolysis	3-Phosphoglycerate	C3H7O7P	11.33	187.00022	184.98566	0.5	0.4	U-^13^C_G6P	X	X	5	5
Glycolysis	Phosphoenolpyruvate (PEP)	C3H5O6P	11.32	168.98965	166.97510	0.1	0.1	U-^13^C_G6P	X	X	0.6	0.08
Glycolysis	Pyruvate	C3H4O3	9.93	89.02332	87.00877	–	3.1	U-^13^C_Glutamate	X	X	–	10
Glycolysis	Lactate	C3H6O3	6.93	91.03897	89.02442	–	1.9	U-^13^C_Glutamate	X	X	–	2.5
Glycolysis	Glucose	C6H12O6	8.24	181.07066	179.05611	–	0.1	U-^13^C_G6P	X	X	–	–
Energy	Creatine	C4H9N3O2	8.6	132.07675	130.06220	0	2	U-^13^C_Glutamine	X	X	0.08	2.5
Energy	Creatinine	C4H7N3O	5.49	114.06619	112.05164	0	0.6	U-^13^C_Glutamine	X	X	0.08	0.15
Energy	Phosphocreatine	C4H10N3O5P	9.85	212.04308	210.02853	0	–	U-^13^C_Glutamine	X	X	–	–
Energy	Adenosine monophosphate (AMP)	C10H14N5O7P	9.13	348.07036	346.05581	0	0.1	U-^13^C_AMP	X	X	0.08	0.08
Energy	Adenosine diphosphate (ADP)	C10H15N5O10P2	10.17	428.03669	426.02214	0	0.1	U-^13^C_AMP	X	X	0.3	0.3
Energy	Adenosine triphosphate (ATP)	C10H16N5O13P3	10.88	508.00302	505.98847	0.1	0.1	U-^13^C_AMP	X	X	0.6	0.3
Nucleotides	Cytidine monophosphate (CMP)	C9H14N3O8P	9.95	324.05913	322.04458	0	–	U-^13^C_AMP	X	X	–	–
Nucleotides	Cytidine diphosphate (CDP)	C9H15N3O11P2	11.01	404.02546	402.01091	0	–	U-^13^C_AMP	X	X	–	–
Nucleotides	Cytidine triphosphate (CTP)	C9H16N3O14P3	11.71	483.99179	481.97724	0	–	U-^13^C_AMP	X	–	–	–
Nucleotides	Guanosine monophosphate (GMP)	C10H14N5O8P	10.39	364.06528	362.05072	0	0.3	U-^13^C_UDP-Glc	X	X	0.08	0.08
Nucleotides	Guanosine diphosphate (GDP)	C10H15N5O11P2	11.53	444.03161	442.01705	0	0.1	U-^13^C_UDP-Glc	X	X	0.6	0.3
Nucleotide	Guanosine triphosphate (GTP)	C10H16N5O14P3	12.17	523.99794	521.98338	0	–	U-^13^C_AMP	X	–	–	–
Nucleotides	Uridine monophosphate (UMP)	C9H13N2O9P	10.38	325.04314	323.02859	0	0	U-^13^C_AMP	X	X	0.15	0.08
Nucleotides	Uridine diphosphate (UDP)	C9H14N2O12P2	12.60	405.00947	402.99492	0.2	–	U-^13^C_AMP	X	X	–	–
Nucleotides	Uridine triphosphate (UTP)	C9H15N2O15P3	11.52	484.97581	482.96125	0	–	U-^13^C_AMP	X	–	–	–
Nucleotides	Inosine monophosphate (IMP)	C10H13N4O8P	11.44	349.05438	347.03982	0.1	0.1	U-^13^C_AMP	X	X	0.3	0.08
Nucleotides	Adenosine diphosphoribose (ADPR)	C15H23N5O14P2	9.60	560.07895	558.06440	0	0.1	U-^13^C_G6P	X	X	0.08	0.08
Nucleotides	Uridine diphosphoglucuronic acid (UDP-glucuronate)	C15H22N2O18P2	15.05	581.04156	579.02701	−0.2	−0.5	U-^13^C_G6P	X	X	2.5	0.08
PPP	Ribose-5-phosphate (R5P)	C5H11O8P	10.78	231.02643	229.01188	0.1	0.3	–	Same peak as Xu5P	X	20	1.25
PPP	Xylulose-5-phosphate (Xu5P)	C5H11O8P	10.49	231.02643	229.01188	0.5	0.5	U-^13^C_G6P	X	X	1.25	0.3
PPP	Sedoheptulose-7-phosphate (SH7P)	C7H15O10P	10.22	291.04756	289.03301	0.3	0.2	U-^13^C_Sedoheptulose-7-phosphate	X	X	5	0.15
Redox	Flavin adenine dinucleotide (FAD^+^)	C27H33N9O15P2	8.11	786.16441	784.14986	0.1	0.1	U-^13^C_Glutamate	X	X	0.08	0.08
Redox	Glutathione (GSH)	C10H17N3O6S	9.08	308.09108	306.07653	0	0.1	U-^13^C_AMP	X	X	0.08	0.15
Redox	Glutathione disulfide (GSSG)	C20H32N6O12S2	10.76	613.15924	611.14469	0	0	U-^13^C_AMP	X	X	0.08	0.08
Redox	Nicotinamide adenine dinucleotide oxidized (NAD^+^)	C21H27N7O14P2	8.98	664.11640	662.10185	0	0.1	U-^13^C_NAD^+^	X	X	0.08	0.08
Redox	Nicotinamide adenine dinucleotide reduced (NADH)	C21H29N7O14P2	9.41	666.13204	664.11750	0	0.2	U-^13^C_NAD^+^	X	X	0.08	0.08
Redox	Nicotinamide adenine dinucleotide phosphate oxidized (NADP^+^)	C21H28N7O17P3	10.81	744.08273	742.06818	0.2	0.1	U-^13^C_NAD^+^	X	X	0.6	0.6
Redox	Nicotinamide adenine dinucleotide phosphate reduced (NADPH)	C21H30N7O17P3	11.11	746.09838	744.08383	0.3	–	U-^13^C_NAD^+^	X	X	–	–
TCA cycle	Acetyl-CoA	C23H38N7O17P3S	8.96	810.13305	808.11850	0.1	0	U-^13^C_G6P	X	X	0.08	0.15
TCA cycle	Citrate	C6H8O7	11.69	193.03428	191.01973	–	0	U-^13^C_Glutamate	X	X	–	0.08
TCA cycle	α-Ketoglutarate	C5H6O5	10.20	147.02880	145.01425	–	0.2	U-^13^C_G6P	X	X	–	0.08
TCA cycle	Succinate	C4H6O4	9.95	119.03389	117.01933	–	0.1	U-^13^C_Glutamate	X	X	–	0.3
TCA cycle	Fumarate	C4H4O4	10.35	117.01824	115.00368	–	0.5	U-^13^C_Glutamate	X	X	–	2.5
TCA cycle	Malate	C4H6O5	10.34	135.02880	133.01425	–	0.1	U-^13^C_Glutamate	X	X	–	0.08
1C metabolism	*S*-Adenosyl-L-homocysteine (SAH)	C14H20N6O5S	8.28	385.12887	383.11431	0.1	0	U-^13^C_Glutamine	X	X	0.08	0.08
1C metabolism	*S*-Adenosyl-L-methionine (SAM)	C15H22N6O5S	10.38	399.14452	397.12996	0.5	-0.3	U-^13^C_AMP	X	X	0.08	10
Heme biosynthesis	5-Aminolevulinic acid	C5H9NO3	8.31	132.06552	130.05097	−0.3	0.7	U-^13^C_G6P	X	X	0.08	1.25
Heme biosynthesis	Porphobilinogen	C10H14N2O4	9.77	227.10263	225.08808	−0.4	0.1	U-^13^C_G6P	X	X	0.6	0.08
Sugar	Uridine diphosphate-glucose (UDP-Glc)	C15H24N2O17P2	10.65	567.06230	565.04774	0.9	0.3	U-^13^C_UDP-Glc	X	X	0.6	0.3
Sugar	Uridine diphosphate N-acetylglucosamine (UDP-GlcNAc)	C17H27N3O17P2	9.72	608.08885	606.07429	0	0.3	U-^13^C_UDP-GlcNAc	X	X	0.08	0.08
Other	Carnitine	C7H15NO3	7.88	162.11247	160.09792	0	4.1	U-^13^C_Glutamine	X	X	0.08	20
Other	Glycerol phosphate	C3H9O6P	9.73	173.02095	171.00640	–	3.7	U-^13^C_Glutamate	X	X	–	–
Other	Phosphocholine	C5H14NO4P	9.39	184.07332	182.05877	0	–	U-^13^C_Glutamine	X	–	–	–
Other	γ-Aminobutyric acid	C4H9NO2	9.03	104.07061	102.05605	0	2.5	U-^13^C_Aminobutyric acid	X	X	0.08	2.5
Other	Pantothenic acid	C9H17NO5	6.45	220.11795	218.10340	0.1	0	U-^13^C_Glutamine	X	X	0.08	0.08
Other	N-Acetylaspartate	C6H9NO5	9.70	176.05535	174.04080	–	1.1	U-^13^C_Glutamate	X	X	–	–
Other	Cystathionine	C7H14N2O4S	10.08	223.07640	221.06184	0.4	–	U-^13^C_AMP	X	X	–	–
Other	Raffinose	C18H32O16	9.83	505.17631	503.16176	0	0.1	–	–	X	0.15	0.08
IS	U-^13^C_Glutamine	^13^C5H10N2O3	9.02	152.09319	–	2.4	–	–	–	–	–	–
IS	U-^13^C_Arginine	^13^C6H14N4O2	15.23	181.13908	–	0.3	–	–	–	–	–	–
IS	U-^13^C_Glutamate	^13^C5H9NO4	9.33	153.07721	151.06266	1.2	0.4	–	–	–	–	–
IS	U-^13^C_AMP	^13^C10H14N5O7P	9.15	358.10391	–	0.2	–	–	–	–	–	–
IS	U-^13^C_NAD^+^	^13^C21H27N7O14P2	9.00	685.18685	–	0.5	–	–	–	–	–	–
IS	U-^13^C_UDP-Glc	^13^C15H24N2O17P2	10.44	–	580.09807	–	1.8	–	–	–	–	–
IS	U-^13^C_G6P	^13^C6H13O9P	10.54	–	265.04257	–	0.5	–	–	–	–	–
IS	U-^13^C_Aminobutyric acid	^13^C4H9NO2	9.27	–	106.06947	–	3.3	–	–	–	–	–
IS	U-^13^C_UDP-GlcNAc	^13^C17H27N3O17P2	9.89	–	623.13133	–	0.1	–	–	–	–	–
IS	U-^13^C_Sedoheptulose-7-phosphate	^13^C7H15O10P	10.22	–	296.05649	–	0.6	–	–	–	–	–

The table displays: pathway or metabolite classification; metabolite common name; chemical formula; RT: retention time (minutes); *m/z* theoretical: [M + H]^+^ for positive ions, while [M − H]^−^ for negative ions; mass error (in ppm, for positive, + , and negative, -, ion modes); ^13^C-internal standard (IS) assigned for data normalization; intracellular presence (if found in cells, x); confirmation of metabolite identification by an authentic standard (ACS); and the metabolite’s limit of detection (LOD, µM) in + , and − ion modes. AA, amino acid; PPP, pentose phosphate pathway; TCA, tricarboxylic acid; redox, involved in redox reactions. See also Fig. S2

### Data analysis

The SMILES, *logP,* and *logD* at pH 9.5 of each authentic standard used in the CCM LC-MS method development were obtained by PubChem (SMILES) and ChemAxon’s Partitioning calculator (*logP* and *logD*) and are reported in Table S2. Statistical analyses were performed using R version 4.2.1. Prior to principal component analysis (PCA), metabolite intensities were normalized using min–max scaling. PCA was then performed using the prcomp function, and the results were visualized with ggplot2. Differential metabolite analysis was conducted between the control and experimental groups using two-sample t-tests on LC-MS intensity (area under the curve) values. *P-*values were corrected for multiple testing with the Benjamini–Hochberg false discovery rate (FDR) method, and metabolites with FDR-adjusted *p*-values < 0.05 were considered significant. The top 10 significant metabolites were visualized as bar plots displaying group means ± standard deviations (SD) with individual sample values overlaid.

## Results and discussion

In this study, we developed a comprehensive analytical pipeline, from acquisition to data visualization, for examining CCM intermediates (mainly consisting of amino acids, carboxylic acids, nucleotides, and sugar phosphates) in cellular extracts using semi-targeted LC-HRMS.

### LC-HRMS CCM metabolite panel construction

First, a panel of  > 77 metabolites from CCM, including side pathways, were selected for analysis. Authentic standards were prepared and acquired using the proposed LC-HRMS analytical method from which seventy-seven metabolites’ chemical properties were determined. A total of 24 amino acids (and derivatives), 9 glycolytic intermediates, 6 energy sources, 9 nucleotides, 3 PPP intermediates, 7 redox metabolites, 6 TCA cycle intermediates, 2 one carbon metabolism intermediates, 2 heme biosynthesis intermediates, 2 sugar donors, and 7 other metabolites were confirmed, Table 1 and Fig. S2.

**Fig. 1:**
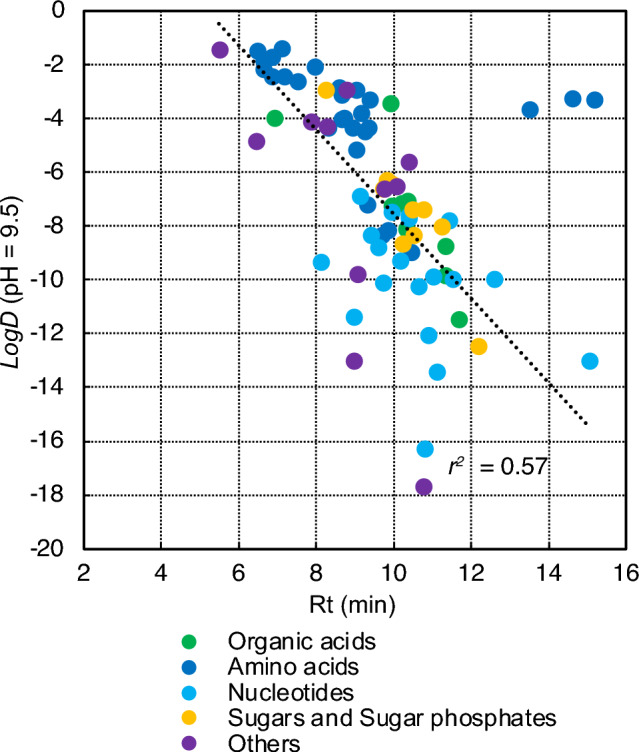
Correlation between retention time (RT) obtained by HILIC chromatography and *logD* for central carbon metabolism intermediates. A total of 77 metabolites analyzed by LC-HRMS method: organic acids (9; green), amino acids and derivatives (28, dark blue), nucleotides and derivatives (19; light blue), sugars and sugar phosphates (10; yellow), and other metabolites (11; purple). Retention time (RT, min) in the x-axis, while *logD* (at pH 9.5) in the y-axis. Data in Table S2

To better understand the capacity of HILIC to separate CCM intermediates, we evaluated the relationship between metabolite polarity and retention time (Fig. 1, Table S2). The *logD* (*logP* corrected for pH) values for all metabolites were obtained. In general, higher metabolite *logD* (higher lipophilicity) led to lower RTs (Fig. 1). The effect of the pH, reflected in the *logD* (and not in the *logP*), was relevant in improving this trend (*r*^2^ ~ 0.57, excluding 4 outliers: amino acids arginine, lysine, and ornithine, and oxidized form of glutathione GSSG). This is in line with previous studies, which elaborated on the correlation between higher compound polarity and a stronger interaction with the stationary phase’s water layer (Buszewski & Noga, 2012; Guo, 2015). However, the correlation between RT and *logD* is not strictly linear, making the study of HILIC separation complex and potentially influenced by other physico-chemical properties. Bonini et al. developed Retip (Bonini et al., 2020), an open source R package that predicts LC (including HILIC) RTs from molecular structures. Retip calculates hundreds of 2D molecular descriptors (such as *logP*, atom counts, and pKa values) from compound SMILES and then uses them as inputs to train five integrated machine learning algorithms—Random Forest, Bayesian-Regularized Neural Network, XGBoost, LightGBM, and Keras—to model the quantitative relationships between molecular structure and experimental RTs. Other approaches, such as GNN-TL (Graph Neural Network with Transfer Learning) (Yang et al., 2021), use advanced deep learning by representing molecules as graphs, thereby learning molecular features via graph neural networks (GNNs), which capture both molecular topological and chemical information. These are then used to correlate with experimental RTs. The GNN model was first trained on a large in silico HILIC RTs dataset and then fine-tuned on an experimental training set (Yang et al., 2021). These advanced tools can further enhance metabolite annotation when new metabolites are to be included within an LC–MS targeted panel.

In CCM, there are several isomers. For example, glucose-6-phosphate and fructose-6-phosphate, with [M-H]^−^ 259.02244 m*/z*, eluted at 10.53 and 11.25 min, respectively, confirmed by authentic standards. Isoleucine and leucine, with [M + H]^+^ 132.10191 m*/z*, eluting at 6.86 and 6.64 min, respectively, remained difficult to separate under the chromatographic conditions, and depending on their concentration, chromatographic resolution may not be possible. The PPP intermediates xylulose-5-phosphate and ribose-5-phosphate, with [M-H]^−^ 229.01188 m*/z*, eluted at 10.49 and 10.78 min, respectively, and are equally poorly resolved under the current chromatographic separation, which imposes limitations on their quantification in biological samples. Distinction between some of these isomers would then profit from additional method development, such as specific fragmentation patterns through MS/MS, feasible to implement in QqQ-MS instruments (Buescher et al., 2010), sequential windowed acquisition of all theoretical fragment ions (SWATH) in HR-MS (Anjo et al., 2017), and/or even the inclusion of alternative fragmentation modes, beyond collision induced dissociation, such as electron activated dissociation (EAD) (Che et al., 2023).

To assess the linearity range of the CCM intermediates in this method, standards were analysed at 9 different concentrations, ranging from 0.08 to 20 µM, and acquired in both positive and negative ion modes by LC-ESI-HRMS. For displaying compound-standard curves (Fig. S3), only concentrations within the linear range, with *r*^2^ > 0.95, were considered, based on a minimum of five concentration points. Pyruvate and glyceraldehyde-3-phosphate did not meet these requirements and thus were not included. Furthermore, the limit of detection (LOD) was identified for each metabolite. For amino acids, positive ion mode resulted in lower LOD, compared to negative ion mode. This is the case for alanine, arginine, and glycine. For the rest of the metabolites (especially for TCA cycle intermediates and sugar phosphates), LOD was lower or equal in negative ion mode (Table 1). The instrument’s dynamic range for all measured compounds was within 2–3 orders of magnitude.

To further complement the metabolite panel, cellular extracts were acquired. Acquisition of intracellular samples was performed to assess the number of metabolites present in real biological extracts by their accurate mass (± 5 ppm), and to identify additional metabolites not confirmed by authentic standards (mostly due to constraints on commercial availability). A total of 76 putative metabolites were found intracellularly, of which 72 were confirmed with authentic standards. Mainly, certain nucleotide forms (e.g., guanosine triphosphate, uridine triphosphate, and cytidine triphosphate) could not be confirmed with authentic standards, due to poor chromatographic behaviour, and thus inability to obtain a consistent RT. In matrix, the LC–MS performance of these metabolites still allowed us to propose a putative identification.

**Fig. 2:**
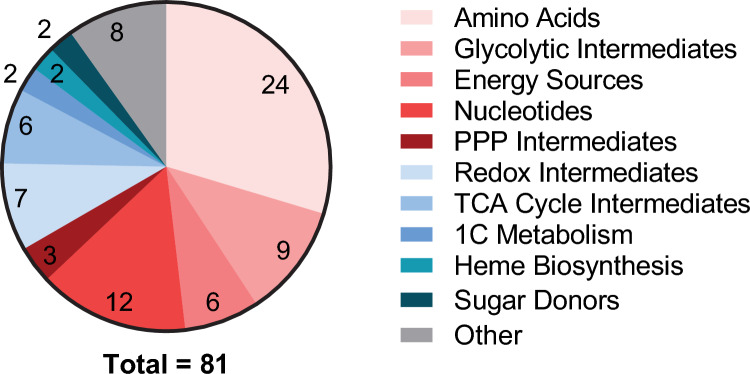
Pathway distribution of metabolites detected by the LC-HRMS central carbon metabolism method. Eighty-one metabolites were monitored (77 confirmed by authentic standards), and the remaining were putatively identified using accurate mass: 24 amino acids, 9 glycolytic intermediates, 6 energy sources, 12 nucleotides, 3 PPP intermediates, 7 redox metabolites, 6 TCA cycle intermediates, 2 one carbon metabolism intermediates, 2 heme biosynthesis intermediates, 2 sugar donors, and 8 other metabolites. 1C, one carbon; PPP, pentose phosphate pathway; TCA, tricarboxylic acid. Data in Table 1, and LC–MS chromatograms in Fig. S2

Therefore, the complete targeted metabolome panel consisted of 81 putative metabolites: 24 amino acids, 9 glycolytic intermediates, 6 energy sources, 12 nucleotides, 3 PPP intermediates, 7 metabolites involved in redox reactions, 6 TCA intermediates, 2 one carbon metabolism intermediates, 2 heme biosynthesis intermediates, 2 sugar donors, and 8 metabolites involved in other metabolic reactions (Fig. 2).

Previous studies reported the identification and quantification of 138 metabolites (sugar phosphates, nucleotides, amino acids, aromatics, and coenzymes, among others) using ion-pairing RP UHPLC coupled with QqQ tandem mass spectrometry (MS/MS) (Buescher et al., 2010). Although our method covered fewer metabolites, the use of HILIC enables the separation of polar compounds without the need for ion-pairing agents. Ion-pairing agents are not ideal for prolonged use in mass spectrometers due to potential contamination and/or interference with other applications, when sharing equipment. HILIC was reported elsewhere as the most appropriate separation technique to perform untargeted CCM profiling compared to RP and MMC (Girel et al., 2023). To increase metabolite coverage, including hydrophobic compounds, a dual separation mass spectrometry with RP and HILIC, previously used in untargeted studies (Ivanisevic et al., 2013), could further be implemented in the here reported method. Bajad and colleagues performed the characterization of 141 CCM metabolites, 69 quantified from *E. coli* extracts acquired by HILIC-QqQ-MS (Bajad et al., 2006). The use of SRM in targeted studies enables the robust and absolute quantification of intended metabolites along a wide linear range. Nevertheless, SRM methods require extensive development (Grund et al., 2016; Lu et al., 2008). With the use of full scan acquisition, method development is faster, making it more easily transferable between instruments. However, absolute quantification in HR-MS, such as TOF instruments, remains challenging due to the narrower dynamic range (Lu et al., 2008).

### ^13^C internal standard for LC–MS signal intensity (area under the curve) normalization

To correct for possible variation occurring during sample preparation and acquisition, metabolite intensities from cellular extracts were corrected with the in-house-generated IS, yeast grown with U-^13^C glucose, effectively containing all intracellular metabolites labelled with ^13^C. To optimize on this resource, while obtaining stable instrumental metabolite intensities, not all U-^13^C-metabolites were selected as IS. From the U-^13^C-labeled metabolites, 11 were chosen to normalize the entire CCM metabolite panel (Tables 1; Fig. S4): ^13^C-adenosine monophosphate (U-^13^C-AMP), U-^13^C-arginine, U-^13^C-glutamate, U-^13^C-glutamine and U-^13^C-nicotinamide adenine dinucleotide (U-^13^C-NAD^+^) for positive ion mode, and U-^13^C-aminobutyric acid, U-^13^C-glucose-6-phosphate (U-^13^C-G6P), U-^13^C-sedoheptulose-7-phosphate (U-^13^C-SH7P), U-^13^C-uridine diphosphate glucose (U-^13^C-UDP-Glc), U-^13^C-uridine diphosphate N-acetylglucosamine (U-^13^C-UDP-GlcNAc), and U-^13^C-glutamate for negative ion mode analyzes. These metabolites were selected due to their stable detection (< 22% average variation to average signal) across samples (Fig. S4). The LC–MS intensity (area under the curve) of targeted CCM metabolites was normalized using one of the 11 U-^13^C-labeled metabolites with the most similar structure and/or the closest retention time. For example, U-^13^C-glutamine was used to normalize all amino acids, while U-^13^C-G6P was used to normalize glycolytic intermediates.

A more refined selection of IS for each metabolite has been proposed with NOMIS (normalization using optimal selection internal standard). This method uses the variability information from multiple IS compounds to find the optimal normalization factor for each individual molecular species (Sysi-Aho et al., 2007).

### Applying CCM LC-HRMS method to study the metabolic effects of a set of mitochondrial inhibitors

Our established LC-HRMS method was challenged to detect changes in cellular CCM metabolite intensities induced by known mitochondrial inhibitors: 2-deoxy-d-glucose (2DG), etomoxir, 3-nitropropionic acid, UK-5099, and rotenone. 2DG acts as a glucose analogue, inhibiting glycolysis (Pajak et al., 2019). Etomoxir impairs fatty acid oxidation as it is an inhibitor of the carnitine palmitoyl-transferase 1a (CPT1a) (O’Connor et al., 2018). 3-Nitropropionic acid inhibits the mitochondrial complex II, affecting succinate dehydrogenase, which metabolizes succinate to fumarate in the TCA cycle (Brouillet et al., 2005). UK-5099 blocks the transport of pyruvate into the mitochondria (Wang et al., 2023). Rotenone is a potent mitochondrial complex I inhibitor that enhances the production of reactive oxygen species (Li et al., 2003).

**Fig. 3:**
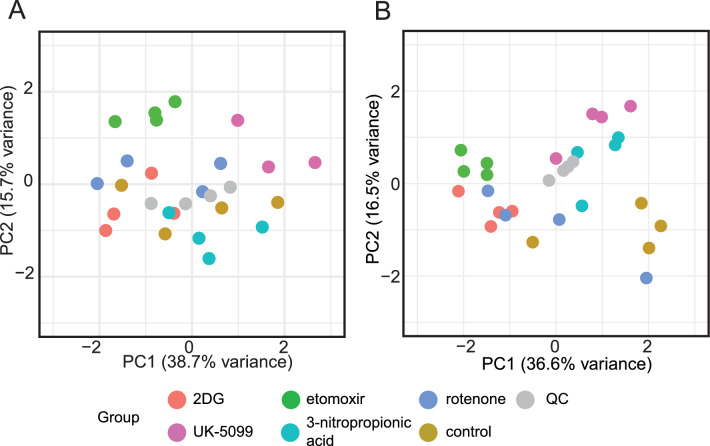
U-^13^C-internal standard (IS) correction contributes to the robustness of LC-HRMS central carbon metabolism data of cellular extracts exposed to 5 mitochondrial inhibitors. Principal component analysis (PCA) scores plots of cells incubated with 2-deoxy-glucose, 2DG (red), etomoxir (green), rotenone (purple), UK-5099 (pink), 3-nitropropionic acid (blue), and control (brown). Quality control samples (QCs, pool of all conditions) are represented in grey. **A** LC-HRMS data without IS normalization. **B** LC-HRMS data normalized by IS. LC-HRMS metabolite intensity (area under the curve) data were normalized using min–max scaling to the [0,1] range prior to PCA; N = 4. Supporting information in Fig. S4

Principal component analysis (PCA) was applied to the obtained LC-HRMS data to assess technical performance of the method on replicate reproducibility and sample type separation (Fig. 3, and Supplementary File 2). Metabolite intensities without (Fig. 3A) and with (Fig. 3B) IS normalization were compared. Samples (cellular extracts incubated with the inhibitors) normalized by the IS presented a clearer group separation and better replicate reproducibility compared to those without IS normalization. This indicated that our IS normalization approach improved result reliability and supported data interpretation. In the normalized results (Fig. 3B), the etomoxir condition was the furthest apart from the control condition. Furthermore, the conditions of UK-5099 and 3-nitropropionic acid appeared proximate to each other in the PCA, which can be explained by the cells’ similar responses to compound exposure. In fact, both inhibitors, UK-5099 and 3-nitropropionic, directly interfere with the TCA cycle since the former reduces pyruvate availability and the latter inhibits succinate dehydrogenase (Brouillet et al., 2005; Wang et al., 2023). Replicates of the rotenone condition appeared spread out across PC1, indicating high biological variability.

These results confirm that our LC-HRMS analytical approach captures changes in metabolite intensities upon perturbation, enabling the generation of robust data suitable for addressing biological questions.

### Biological interpretation of mammalian cells incubated with CCM inhibitors

**Fig. 4:**
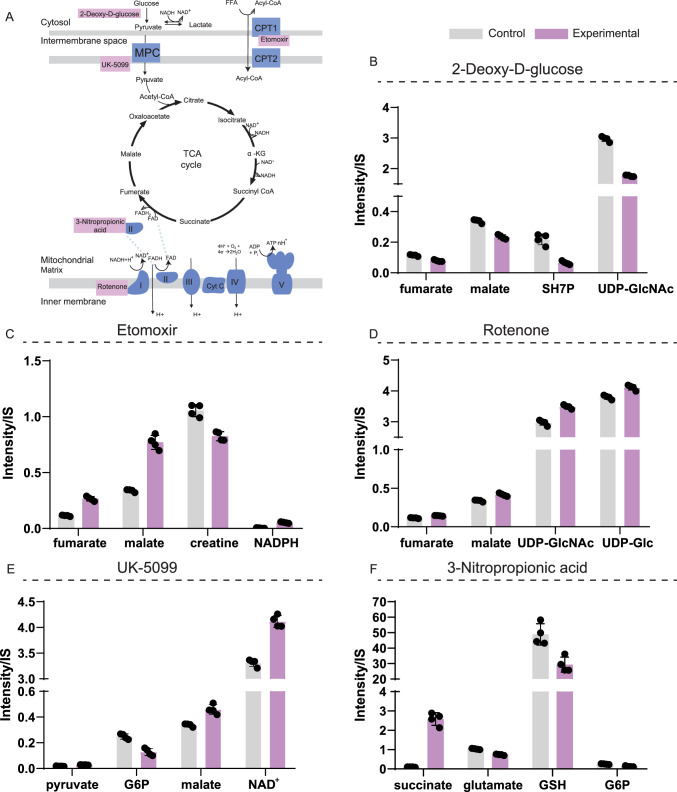
Metabolic changes within Central Carbon Metabolism, induced by 5 inhibitors on mammalian cells. **A** Simplified scheme depicting the mechanism of action of the 5 inhibitors used: 2-deoxy-d-glucose, etomoxir, rotenone, UK-5099, and 3-nitropropionic acid on CCM. **B–F** Normalized LC-HRMS intensities of affected metabolites, in control (grey) and experimental (purple) conditions. **B** 2-Deoxy-d-glucose, **C** Etomoxir, **D** Rotenone, **E** UK-5099, **F** 3-Nitropropionic acid. LC-HRMS intensity (area under the curve) of each metabolite was divided by the IS; N = 4 ± SD. All bar-plot comparisons, between control and experimental conditions, are statistically significant (FDR-adjusted *p* < 0.05; two-sample t-test; except for fumarate in the rotenone panel, FDR-adjusted *p-value* = 0.069). MPC, mitochondrial pyruvate carrier; CPT1, carnitine palmitoyltransferase 1; CPT2, carnitine palmitoyltransferase 2; NAD(H), nicotinamide adenine dinucleotide; FAD(H_2_), flavin adenine dinucleotide; TCA, tricarboxylic acid; α-KG, α-ketoglutarate; FFA, free fatty acid. Supporting information in Figs. S5–9 and Table S3

We further examined the specific metabolic changes induced by mitochondrial inhibitors in the cell system used. Univariate statistics using a t-test were performed to compare metabolite abundances obtained by LC-HRMS between control and experimental conditions. The top 10 metabolites with the lowest significant adjusted *p*-value were displayed as barplots for each experimental condition (Figs. S5–9). Variations in some of these metabolites, due to inhibitor exposure, were further discussed (Fig. 4, and Table S3).

2DG (Fig. 4B) led to a reduction in the level of TCA cycle intermediates (fumarate and malate), as a consequence of an impaired glycolytic flux. Other glucose-dependent pathways were downregulated, including the PPP (Patra & Hay, 2014), resulting in low levels of SH7P, and the hexosamine biosynthetic pathway (Lam et al., 2021), which was noted for the reduced levels of UDP-GlcNAc. Interestingly, none of the glycolytic intermediates measured (e.g., G6P or pyruvate) were among the top 10 metabolites with the lowest *p*-values, despite the evident glucose accumulation that resulted from the inhibition (Fig. S5).

Cells incubated with etomoxir (Figs. 4C, S6) presented a higher activity of the TCA cycle, as evidenced by fumarate and malate accumulation. Higher TCA cycle intermediates can result from a compensatory mechanism that generates ATP via alternative carbon sources, as acetyl-CoA incorporation from free fatty acid (FFA) oxidation is inhibited. This is also consistent with lower creatine levels (Deminice et al., 2016). Elevated levels of malate may explain high levels of NADPH, obtained through malate conversion to pyruvate, a reaction carried out by the malic enzyme (Y.-P. Wang et al., 2021), part of the pyruvate-malate shuttle (MacDonald, 1995). Moreover, the inhibition of fatty acid oxidation may increase glycolytic flux, thereby shunting more G6P into the PPP and leading to further NADPH production.

Rotenone (Figs. 4D, S7), a powerful mitochondrial complex I inhibitor (Li et al., 2003), significantly increased TCA cycle intermediates (higher fumarate and malate abundance). This underscores a compensatory mechanism of the cell in response to rotenone inhibition, enabling energy production. Under these conditions, glucose-dependent pathways such as the hexosamine biosynthetic pathway and glycogen biosynthesis were upregulated (resulting in higher levels of UDP-GlcNAc and UDP-Glc).

Cells incubated with UK-5099 (Figs. 4E, S8) showed an accumulation of pyruvate. High pyruvate levels do not directly inhibit glycolysis, but can lower the glycolytic downstream (Dyrstad et al., 2021) resulting in lower G6P production. An increase in malate was observed, probably through the pyruvate-malate shuttle (MacDonald, 1995; Martínez-Reyes & Chandel, 2020; Y.-P. Wang et al., 2021). Furthermore, high levels of NAD^+^ can be explained by higher lactate dehydrogenase activity, to remove excess pyruvate (Lin et al., 2022).

Last, when cells were incubated with 3-nitropropionic acid (Figs. 4F, S9), an inhibitor of the succinate dehydrogenase (Brouillet et al., 2005), the substrate of the reaction (succinate) was accumulated. TCA cycle intermediates were therefore reduced, leading to lower glutamate amounts. GSH levels were reduced, likely due to decreased reducing power from TCA cycle inhibition. Lastly, an increase in G6P consumption was observed, indicating higher glucose utilization as a compensatory mechanism.

## Conclusion and outlook

We report here a HILIC-HR-TOF-MS metabolome method covering 82

CCM metabolites, mostly with unambiguous identification. The analytical properties of the metabolites within this method were described, and linear instrumental responses enabled LOD determination for 63 of these compounds. Whilst chromatographic performance remains challenging for phosphate-containing species, in cellular extracts, 78 metabolites were putatively identified (being 74 confirmed with authentic standards). The method was used to showcase the metabolic effects of mitochondrial inhibitors on central metabolite levels (2DG, etomoxir, rotenone, UK-5099, and 3-nitropropionic acid). LC–MS data normalization using U-^13^C-yeast extract as internal standard was an effective strategy to improve method performance.

In conclusion, the HILIC-HR-TOF-MS metabolome method reported here is suitable for the relative quantification of CCM metabolites, facilitating data interpretation to answer a wide variety of research questions. Ultimately, this method may serve as a starting point for future research by expanding metabolite coverage and applying it to a variety of biological samples, e.g., biofluids, tissues, bacteria, and plants.

## Supplementary Information

Below is the link to the electronic supplementary material.

Supplementary file1 (PDF 4812 KB)

Supplementary file2 (XLSX 27 KB)

## Data Availability

Data of this study are available in the Supplementary Materials. Data for the LC–MS dataset shown in Fig. 3 are provided as a spreadsheet (Supplementary File 2).
