# Supplementary material for: Scanning central carbon metabolism: a HILIC-HR-TOF-MS metabolome method
Source: Metabolomics. 2026 May 13;22(3):71. doi: 10.1007/s11306-026-02434-4 (PMC13171983; doi:10.1007/s11306-026-02434-4)
Supplement: Supplementary file 1 — Supplementary file1 (PDF 4812 KB) [file 11306_2026_2434_MOESM1_ESM.pdf]

Supplementary Information

Scanning Central Carbon Metabolism: a HILIC-HR-TOF-MS metabolome method

Victoria Pozo Garcia[1], Valentina Ferro[1], Jolene Rier[1], Sofia Moco[1]*

[1] Department of Chemistry and Pharmaceutical Sciences, Amsterdam Institute of Molecular and Life Sciences (AIMMS), Vrije Universiteit Amsterdam, Amsterdam, the Netherlands

*Corresponding author: s.moco@vu.nl

Keywords: LC-MS, metabolomics, central carbon metabolism, HILIC

SUPPLEMENTARY TABLES

Table S1 Authentic standards used for LC-MS method development of CCM. Chemical name, with corresponding biochemical acronym in parenthesis

Chemical	Supplier	CAS number	Stock concentration (mM)
5-Aminolevulinic acid hydrochloride (ALA)	Apollo Scientific	5451-09-2	10
Acetyl coenzyme A lithium salt (Acetyl-CoA)	Sigma	32140-51-5	10
Adenosine 5′-diphosphoribose sodium salt	Sigma	68414-18-6	10
Adenosine 5′-diphosphate sodium salt (ADP)	Sigma	20398-34-9	10
Adenosine 5′-monophosphate disodium salt (AMP)	Sigma	4578-31-8	10
Adenosine 5′-triphosphate disodium salt hydrate (ATP)	Sigma	34369-07-8	10
L-Alanine	Sigma	56-41-7	10
L-Arginine	Sigma	74-79-3	10
L-Asparagine	Sigma	70-47-3	10
L-Aspartic acid	Sigma	56-84-8	5
L-Carnitine hydrochloride	Sigma	6645-46-1	10
Citric acid	Sigma	77-92-9	10
L-Citrulline	Sigma	372-75-8	10
Creatine	Sigma	57-00-1	10
Creatinine	Sigma	60-27-5	10
L-Cystathionine	Sigma	56-88-2	10
Cytidine-5'-triphosphate disodium (CTP)	BLDpharm	36051-68-0	10
Flavin adenine dinucleotide disodium salt (FAD)	Alfa Aesar	84366-81-4	10
D-Fructose 1,6-bisphosphate trisodium salt hydrate (FBP)	Sigma	38099-82-0	10
D-Fructose 6-phosphate dipotassium salt (F6P)	Sigma	103213-47-4	10
Fumaric acid	Sigma	110-17-8	5
D-(+)-Glucose	Sigma	50-99-7	10
D-Glucose 6-phosphate sodium salt (G6P)	Sigma	54010-71-8	10
L-Glutamic acid potassium salt monohydrate	Sigma	6382-01-0	10
L-Glutamine	Sigma	56-85-9	10
L-Glutathione reduced (GSH)	Sigma	70-18-8	10
L-Glutathione oxidized (GSSG)	Acros organics	27025-41-8	10
Glyceraldehyde-3-phosphate solution (G3P)	Sigma	591-57-1	10
DL-α-Glycerophosphate disodium salt hydrate (GlyP)	Sigma	1555-56-2	10
Glycine	Sigma	56-40-6	10
Guanosine 5′-diphosphate disodium salt (GDP)	Sigma	7415-69-2	10
Guanosine 5′-monophosphate disodium salt hydrate (GMP)	Sigma	5550-12-9	10
L-Histidine	Sigma	71-00-1	10
Inosine 5′-monophosphate disodium salt hydrate (IMP)	Sigma	352195-40-5	10
L-Isoleucine	Sigma	73-32-5	10
L-Kynurenine	BLDpharm	2922-83-0	10
L-Ornithine monohydrochloride	Fluka	3184-13-2	10
Lithium lactate	Sigma	867-55-0	10
L-Leucine	Sigma	61-90-5	10
L-Lysine	Sigma	56-87-1	10
DL-Malic acid	Sigma	6915-15-7	10
L-Methionine	Sigma	63-68-3	10
N-Acetyl-L-aspartic acid	Santa Cruz Biotechnology	997-55-7	10
β-Nicotinamide adenine dinucleotide sodium salt (NAD^+)	Sigma	20111-18-6	10

β-Nicotinamide adenine dinucleotide, reduced disodium salt (NADH)	Sigma	606-68-8	10
β-Nicotinamide adenine dinucleotide phosphate hydrate ($NADP^+$)	Prozomix	53-59-8	10
NADPH tetrasodium salt (NADPH)	Roche	2646-71-1	10
O-Phospho-DL-serine	Sigma	17885-08-4	10
D-Pantothenic acid hemicalcium salt	Sigma	137-08-6	10
L-Phenylalanine	Janssen	63-91-2	10
Phosphocreatine disodium salt hydrate	Sigma	19333-65-4	10
Phospho(enol)pyruvic acid monopotassium salt (PEP)	Roche	138-08-9	10
D-(-)-3-Phosphoglyceric acid disodium salt (3PG)	Sigma	80731-10-8	10
Porphobilinogen monohydrate	MP Biomedicals	487-90-1	0.3
L-Proline	Sigma	147-85-3	10
Pyruvic acid	Sigma	127-17-3	10
D-(+)-Raffinose pentahydrate	Sigma	17629-30-0	10
Ribose 5-phosphate disodium salt hydrate (R5P)	Sigma	207671-46-3	10
S-(5′-Adenosyl)-L-homocysteine (SAH)	Sigma	979-92-0	1
S-(5′-Adenosyl)-L-methionine iodide (SAM)	Sigma	3493-13-8	10
Sedoheptulose 7-phosphate lithium salt	Sigma	2646-35-7	10
L-Serine	Sigma	56-45-1	10
Succinic acid	Sigma	110-15-6	10
Taurine	Sigma	107-35-7	10
L-Threonine	Sigma	72-19-5	10
L-Tryptophan	Thermo Fisher	73-22-3	10
L-Tyrosine	Sigma	60-18-4	1
Uridine 5′-diphosphoglucuronic acid trisodium salt	Sigma	63700-19-6	10
Uridine 5′-diphospho-*N*-acetylglucosamine sodium salt	Sigma	91183-98-1	10
Uridine 5′-diphosphoglucose disodium salt	Sigma	28053-08-9	10
Uridine 5′-monophosphate disodium salt (UMP)	Sigma	3387-36-8	10
Uridine 5′-triphosphate trisodium salt hydrate (UTP)	Sigma	19817-92-6	10
DL-Valine	Sigma	516-06-3	10
Xylulose-5-phosphate lithium salt (Xy5P)	Sigma	2080295-99-2	10
α-Ketoglutaric acid (α-KG)	Sigma	328-50-7	10
γ-Aminobutyric acid	Sigma	56-12-2	10

Table S2 Chromatographic properties of Central Carbon metabolism intermediates. The table displays: chemical classification of the metabolite; metabolite common name; chemical formula; RT: retention time (minutes); compound SMILES; and metabolite *logP* and *logD* at pH 9.5

Class	Compound common name	Chemical formula	SMILES	logP	logD at pH 9.5	RT (min)
Amino acids	Glutamine	C5H10N2O3	C(CC(=O)N)[C@@H](C(=O)O)N	-4	-4.38	8.93
Amino acids	Alanine	C3H7NO2	C[C@@H](C(=O)O)N	-2.84	-3.13	8.66
Amino acids	Arginine	C6H14N4O2	C(C[C@@H](C(=O)O)N)CN=C(N)N	-3.21	-3.34	15.19
Amino acids	Aspartate	C4H7NO4	C([C@@H](C(=O)O)N)C(=O)O	-3.56	-7.24	9.32
Amino acids	Glutamate	C5H9NO4	C(CC(=O)O)[C@@H](C(=O)O)N	-3.21	-3.34	9.38
Amino acids	Glycine	C2H5NO2	C(C(=O)O)N	-3.41	-3.82	9.16
Amino acids	Histidine	C6H9N3O2	C1=C(NC=N1)C[C@@H](C(=O)O)N	-3.6	-4	8.73
Amino acids	Isoleucine	C6H13NO2	CC[C@H](C)[C@@H](C(=O)O)N	-1.51	-1.75	6.86
Amino acids	Leucine	C6H13NO2	CC(C)C[C@@H](C(=O)O)N	-1.59	-1.85	6.64
Amino acids	Lysine	C6H14N2O2	C(CCN)C[C@@H](C(=O)O)N	-3.21	-3.29	14.62
Amino acids	Methionine	C5H11NO2S	CSCC[C@@H](C(=O)O)N	-2.19	-2.46	7.18
Amino acids	Phenylalanine	C9H11NO2	C1=CC=C(C=C1)C[C@@H](C(=O)O)N	-1.18	-1.49	6.48
Amino acids	Proline	C5H9NO2	C1C[C@H](NC1)C(=O)O	-2.57	-2.63	7.52
Amino acids	Serine	C3H7NO3	C([C@@H](C(=O)O)N)O	-3.89	-4.51	9.27
Amino acids	Threonine	C4H9NO3	C[C@H]([C@@H](C(=O)O)N)O	-3.47	-4.05	8.65
Amino acids	Tyrosine	C9H11NO3	C1=CC(=CC=C1C[C@@H](C(=O)O)N)O	-1.49	-2.12	7.95
Amino acids	Valine	C5H11NO2	CC(C)[C@@H](C(=O)O)N	-1.95	-2.19	6.63
Amino acids	Tryptophan	C11H12N2O2	C1=CC=C2C(=C1)C(=CN2)C[C@@H](C(=O)O)N	-1.09	-1.42	7.12
Amino acids	Asparagine	C4H8N2O3	C([C@@H](C(=O)O)N)C(=O)N	-4.29	-5.18	9.05
Amino acids	Citrulline	C6H13N3O3	C(C[C@@H](C(=O)O)N)CNC(=O)N	-3.93	-4.36	9.36
Amino acids	Creatine	C4H9N3O2	CN(CC(=O)O)C(=N)N	-2.86	-2.86	8.6
Amino acids	5-Aminolevulinic acid	C5H9NO3	C(CC(=O)O)C(=O)CN	-3.25	-4.38	8.31
Amino acids	γ-Aminobutyric acid	C4H9NO2	C(CC(=O)O)CN	-2.89	-2.96	9.03
Amino acids	Ornithine	C5H12N2O2	C(C[C@@H](C(=O)O)N)CN	-3.66	-3.69	13.51

Amino acids	N-Acetylaspartate	C6H9NO5	CC(=O)N[C@@H](CC(=O)O)C(=O)O	-1.40	-8.37	9.7
Amino acids	Phosphoserine	C3H8NO6P	C([C@@H](C(=O)O)N)OP(=O)(O)O	-2.85	-9.00	10.44
Amino acids	Phosphocreatine	C4H10N3O5P	CN(CC(=O)O)/C(=N/P(=O)(O)O)/N	-2.25	-8.18	9.85
Amino acids	Kynurenine	C10H12N2O3	C1=CC=C(C(=C1)C(=O)C[C@@H](C(=O)O)N)N	0.37	-2.46	6.85
Sugar phosphates	Glucose-6-phosphate (G6P)	C6H13O9P	C([C@H]([C@H]([C@@H]([C@H](C=O)O)O)O)O)OP(=O)(O)O	-3.69	-8.35	10.53
Sugar phosphates	Fructose-6-phosphate (F6P)	C6H13O9P	C([C@H]([C@H]([C@@H](C(=O)CO)O)O)O)OP(=O)(O)O	-3.39	-8.05	11.25
Sugar phosphates	Fructose-1,6-bisphosphate (F1,6BP)	C6H14O12P2	C([C@@H]1[C@H]([C@@H]([C@@](O1)(COP(=O)(O)O)O)O)O)OP(=O)(O)O	-3.01	-12.47	12.2
Sugar phosphates	Ribose-5-phosphate (R5P)	C5H11O8P	C([C@H]([C@H](C(=O)CO)O)O)OP(=O)(O)O	-2.76	-7.42	10.78
Sugar phosphates	Xylulose-5-phosphate (Xu5P)	C5H11O8P	C([C@H]([C@@H](C(=O)CO)O)O)OP(=O)(O)O	-2.76	-7.42	10.49
Sugar phosphates	Sedoheptulose-7-phosphate (SH7P)	C7H15O10P	C([C@H]([C@H]([C@H]([C@@H](C(=O)CO)O)O)O)O)OP(=O)(O)O	-4.02	-8.68	10.22
Sugar phosphates	Glyceraldehyde-3-phosphate (G3P)	C3H7O6P	C([C@H](C=O)O)OP(=O)([O-])[O-]	-1.8	-6.47	9.94
Sugar phosphates	Glycerol-3-phosphate	C3H9O6P	C(C(COP(=O)(O)O)O)O	-1.96	-6.62	9.73
Sugars	Glucose	C6H12O6	C([C@@H]1[C@H]([C@@H]([C@H](C(O1)O)O)O)O)O	-2.93	-2.94	8.24
Sugars	Raffinose	C18H32O16	C([C@@H]1[C@@H]([C@@H]([C@H]([C@H](O1)OC[C@@H]2[C@H]([C@@H]([C@H]([C@H](O2)O[C@]3([C@H]([C@@H]([C@H](O3)CO)O)O)CO)O)O)O)O)O)O)O	-6.3	-6.3	9.83
Organic acids	3-Phosphoglycerate	C3H7O7P	C(C(C(=O)O)O)OP(=O)(O)O	-1.64	-9.83	11.33
Organic acids	Phosphoenolpyruvate (PEP)	C3H5O6P	C=C(C(=O)O)OP(=O)(O)O	-0.64	-8.75	11.32
Organic acids	Pyruvate	C3H4O3	CC(=O)C(=O)[O-]	0.07	-3.46	9.93
Organic acids	Lactate	C3H6O3	CC(C(=O)[O-])O	-0.47	-4	6.93
Organic acids	Citrate	C6H8O7	C(C(=O)O)C(CC(=O)O)(C(=O)O)O	-1.32	-11.48	11.69
Organic acids	α-Ketoglutarate	C5H6O5	C(CC(=O)O)C(=O)C(=O)O	-0.11	-7.17	10.2
Organic acids	Succinate	C4H6O4	C(CC(=O)O)C(=O)O	-0.4	-7.28	9.95
Organic acids	Fumarate	C4H4O4	C(=C/C(=O)O)\C(=O)O	-0.04	-7.08	10.35
Organic acids	Malate	C4H6O5	C(C(C(=O)O)O)C(=O)O	-1.11	-8.12	10.34
Nucleotides	Adenosine monophosphate (AMP)	C10H14N5O7P	C1=NC(=C2C(=N1)N(C=N2)[C@H]3[C@@H]([C@@H]([C@H](O3)COP(=O)(O)O)O)O)N	-5.06	-6.91	9.13

Nucleotides	Adenosine diphosphate (ADP)	C10H15N5O10P2	C1=NC(=C2C(=N1)N(C=N2)[C@H]3[C@@H]([C@@H]([C@H](O3)COP(=O)(O)OP(=O)(O)O)O)O)N	-4.95	-9.29	10.17
Nucleotides	Adenosine triphosphate (ATP)	C10H16N5O13P3	C1=NC(=C2C(=N1)N(C=N2)[C@H]3[C@@H]([C@@H]([C@H](O3)COP(=O)(O)OP(=O)(O)OP(=O)(O)O)O)O)N	-5.97	-12.09	10.88
Nucleotides	Cytidine diphosphate (CDP)	C9H15N3O11P2	C1=CN(C(=O)N=C1N)[C@H]2[C@@H]([C@@H]([C@H](O2)COP(=O)(O)OP(=O)(O)O)O)O	-2.75	-9.89	11.01
Nucleotides	Cytidine monophosphate (CMP)	C9H14N3O8P	C1=CN(C(=O)N=C1N)[C@H]2[C@@H]([C@@H]([C@H](O2)COP(=O)(O)O)O)O	-2.81	-7.51	9.95
Nucleotides	Guanosine monophosphate (GMP)	C10H14N5O8P	C1=NC2=C(N1[C@H]3[C@@H]([C@@H]([C@H](O3)COP(=O)(O)O)O)O)N=C(NC2=O)N	-2.7	-7.61	10.39
Nucleotides	Guanosine diphosphate (GDP)	C10H15N5O11P2	C1=NC2=C(N1[C@H]3[C@@H]([C@@H]([C@H](O3)COP(=O)(O)OP(=O)(O)O)O)O)N=C(NC2=O)N	-3.24	-9.98	11.53
Nucleotides	Uridine monophosphate (UMP)	C9H13N2O9P	C1=CN(C(=O)NC1=O)[C@H]2[C@@H]([C@@H]([C@H](O2)COP(=O)(O)O)O)O	-2.54	-7.74	10.38
Nucleotides	Inosine monophosphate (IMP)	C10H13N4O8P	C1=NC2=C(C(=O)N1)N=CN2[C@H]3[C@@H]([C@@H]([C@H](O3)COP(=O)(O)O)O)O	-3.06	-7.82	11.44
Nucleotides	Adenosine diphosphoribose	C15H23N5O14P2	C1=NC(=C2C(=N1)N(C=N2)C3C(C(C(O3)COP(=O)(O)OP(=O)(O)OCC4C(C(C(O4)O)O)O)O)O)N	-6.33	-8.8	9.6
Nucleotides	Uridine diphosphoglucuronic acid	C15H22N2O18P2	C1=CN(C(=O)NC1=O)[C@H]2[C@@H]([C@@H]([C@H](O2)COP(=O)(O)OP(=O)(O)O[C@@H]3[C@@H]([C@H]([C@@H]([C@H](O3)C(=O)O)O)O)O)O)O	-4.31	-13.01	15.05
Nucleotides	Flavin adenine dinucleotide (FAD$^+$)	C27H33N9O15P2	CC1=CC2=C(C=C1C)N(C3=NC(=O)NC(=O)C3=N2)C[C@@H]([C@@H]([C@@H](COP(=O)(O)OP(=O)(O)OC[C@@H]4[C@H]([C@H]([C@@H](O4)N5C=NC6=C(N=CN=C65)N)O)O)O)O)O	-5.28	-9.34	8.11
Nucleotides	Nicotinamide adenine dinucleotide oxidized (NAD$^+$)	C21H27N7O14P2	C1=CC(=C[N+](=C1)[C@H]2[C@@H]([C@@H]([C@H](O2)COP(=O)([O-])OP(=O)(O)OC[C@@H]3[C@H]([C@H]([C@@H](O3)N4C=NC5=C(N=CN=C54)N)O)O)O)O)C(=O)N	-10.1	-11.41	8.98
Nucleotides	Nicotinamide adenine dinucleotide phosphate oxidized (NADP$^+$)	C21H28N7O17P3	C1=CC(=C[N+](=C1)[C@H]2[C@@H]([C@@H]([C@H](O2)COP(=O)([O-])OP(=O)(O)OC[C@@H]3[C@H]([C@H]([C@@H](O3)N4C=NC5=C(N=CN=C54)N)OP(=O)(O)O)O)O)O)C(=O)N	-10.5	-16.27	10.81
Nucleotides	Nicotinamide adenine dinucleotide phosphate reduced (NADPH)	C21H30N7O17P3	C1C=CN(C=C1C(=O)N)[C@H]2[C@@H]([C@@H]([C@H](O2)COP(=O)(O)OP(=O)(O)OC[C@@H]3[C@H]([C@H]([C@@H](O3)N4C=NC5=C(N=CN=C54)N)OP(=O)(O)O)O)O)O	-6.42	-13.45	11.11

Nucleotides	Nicotinamide adenine dinucleotide reduced (NADH)	C21H29N7O14P2	C1C=CN(C=C1C(=O)N)[C@H]2[C@@H]([C@@H]([C@H](O2)COP(=O)(O)OP(=O)(O)OC[C@@H]3[C@H]([C@H]([C@@H](O3)N4C=NC5=C(N=CN=C54)N)O)O)O)O	-3.6	-8.36	9.41
Nucleotides	Uridine diphosphate-glucose (UDP-Glc)	C15H24N2O17P2	C1=CN(C(=O)NC1=O)[C@H]2[C@@H]([C@@H]([C@H](O2)COP(=O)(O)OP(=O)(O)O[C@@H]3[C@@H]([C@H]([C@@H]([C@H](O3)CO)O)O)O)O)O	-5	-10.25	10.65
Nucleotides	Uridine diphosphate N-acetylglucosamine (UDP-GlcNAc)	C17H27N3O17P2	CC(=O)N[C@@H]1[C@H]([C@@H]([C@H](O[C@@H]1OP(=O)(O)OP(=O)(O)OC[C@@H]2[C@H]([C@H]([C@@H](O2)N3C=CC(=O)NC3=O)O)O)CO)O)O	-4.92	-10.12	9.72
Nucleotides	Uridine diphosphate (UDP)	C9H14N2O12P2	C1=CN(C(=O)NC1=O)[C@H]2[C@@H]([C@@H]([C@H](O2)COP(=O)(O)OP(=O)(O)O)O)O	-2.85	-10	12.6
Others	Creatinine	C4H7N3O	CN1CC(=O)N=C1N	-1.46	-1.47	5.49
Others	Porphobilinogen	C10H14N2O4	C1=C(C(=C(N1)CN)CC(=O)O)CCC(=O)O	-2.68	-6.64	9.77
Others	Carnitine	C7H15NO3	C[N+](C)(C)C[C@@H](CC(=O)[O-])O	-4.89	-4.12	7.88
Others	Pantothenic acid	C9H17NO5	CC(C)(CO)[C@H](C(=O)NCCC(=O)O)O	-1.36	-4.88	6.45
Others	Taurine	C2H7NO3S	C(CS(=O)(=O)O)N	-2.61	-2.98	8.77
Others	Glutathione (GSH)	C10H17N3O6S	C(CC(=O)N[C@@H](CS)C(=O)NCC(=O)O)[C@@H](C(=O)O)N	-5.04	-9.79	9.08
Others	Glutathione disulphide (GSSG)	C20H32N6O12S2	C(CC(=O)N[C@@H](CSSC[C@@H](C(=O)NCC(=O)O)NC(=O)CC[C@@H](C(=O)O)N)C(=O)NCC(=O)O)[C@@H](C(=O)O)N	-10.2	-17.69	10.76
Others	S-Adenosyl-L-homocysteine (SAH)	C14H20N6O5S	C1=NC(=C2C(=N1)N(C=N2)[C@H]3[C@@H]([C@@H]([C@H](O3)CSCC[C@@H](C(=O)O)N)O)O)N	-4.02	-4.3	8.28
Others	S-Adenosyl-L-methionine (SAM)	C15H22N6O5S	C[S+](CC[C@@H](C(=O)[O-])N)C[C@@H]1[C@H]([C@H]([C@@H](O1)N2C=NC3=C(N=CN=C32)N)O)O	-5.32	-5.64	10.38
Others	Acetyl-CoA	C23H38N7O17P3S	CC(=O)SCCNC(=O)CCNC(=O)[C@@H](C(C)(C)COP(=O)(O)OP(=O)(O)OC[C@@H]1[C@H]([C@H]([C@@H](O1)N2C=NC3=C(N=CN=C32)N)O)OP(=O)(O)O)O	-5.92	-13.04	8.96
Others	Cystathionine	C7H14N2O4S	C(CSCC(C(=O)O)N)C(C(=O)O)N	-1.28	-6.54	10.08

Table S3 Calculated fold changes of LC-MS intensities of CCM intermediates challenged to 2-deoxy-D-glucose, etomoxir, 3-nitropropionic acid, rotenone, and UK-5099, relative to control conditions, with adjusted *p-values* by false discovery rate (FDR) per condition (HepaRG cells incubated over 24 h)

	2-deoxy-D-glucose		Etomoxir		3-nitropropionic acid		Rotenone		UK-5099	
	Ratio to control	adjusted *p-value*	Ratio to control	adjusted *p-value*	Ratio to control	adjusted *p-value*	Ratio to control	adjusted *p-value*	Ratio to control	adjusted *p-value*
(iso)leucine	0.575	0.158	0.530	0.110	1.151	0.714	0.710	0.555	0.861	0.650
ADP	0.648	0.025	0.565	0.011	0.760	0.119	0.923	0.905	0.715	0.064
alanine	0.849	0.327	0.638	0.087	1.119	0.664	0.936	0.822	1.367	0.094
AMP	0.640	0.033	0.437	0.011	0.975	0.889	0.801	0.627	0.714	0.085
arginine	0.781	0.325	0.586	0.110	0.918	0.812	0.665	0.404	0.846	0.603
asparagine	0.732	0.093	0.751	0.104	0.989	0.971	0.792	0.367	1.042	0.771
aspartate	0.405	0.029	1.444	0.040	1.128	0.621	0.900	0.712	2.015	0.010
ATP	0.850	0.189	0.752	0.081	0.683	0.192	1.049	0.928	0.700	0.085
carnitine	0.906	0.364	0.954	0.693	1.266	0.180	1.135	0.627	1.004	0.969
CDP	0.655	0.032	0.608	0.022	0.913	0.630	0.994	0.983	0.970	0.780
cystathionine	0.371	0.003	0.353	0.003	0.633	0.023	0.828	0.569	0.595	0.012
CMP	0.700	0.033	0.586	0.013	0.746	0.112	1.016	0.968	0.807	0.103
creatine	0.911	0.140	0.783	0.006	1.383	0.010	1.356	0.166	1.723	0.056
creatinine	0.680	0.072	0.868	0.247	1.095	0.714	1.063	0.919	1.612	0.121
CTP	0.836	0.158	0.837	0.160	0.691	0.212	1.083	0.905	0.905	0.650
glutamate	0.525	0.000	0.668	0.000	0.715	0.001	0.963	0.456	0.933	0.067
glutamine	0.748	0.136	0.521	0.041	0.993	0.971	0.723	0.324	0.793	0.210
GSH	0.354	0.018	0.561	0.021	0.600	0.034	1.069	0.822	0.592	0.032
GSSG	0.466	0.043	0.455	0.045	0.518	0.112	0.742	0.586	0.465	0.063
GTP	0.868	0.356	0.712	0.105	0.649	0.192	0.951	0.928	0.757	0.258
histidine	0.822	0.361	0.539	0.087	0.990	0.971	0.733	0.485	0.887	0.650
IMP	0.603	0.037	0.437	0.018	0.698	0.122	0.857	0.736	0.556	0.048
kynurenine	0.409	0.043	1.412	0.099	1.005	0.971	0.723	0.367	0.770	0.299

lysine	0.564	0.158	0.493	0.105	0.804	0.664	0.560	0.367	0.792	0.527
methionine	0.592	0.155	0.566	0.110	0.932	0.854	0.667	0.426	0.956	0.859
NAD^+	1.069	0.032	1.272	0.005	1.074	0.034	1.160	0.003	1.243	0.003
NADH	0.984	0.933	1.258	0.105	1.226	0.192	1.145	0.555	1.032	0.822
$NADP^+$	0.860	0.208	0.795	0.050	0.976	0.854	0.870	0.592	1.012	0.896
NADPH	3.938	0.096	13.125	0.002	2.500	0.243	4.625	0.367	4.500	0.013
ornithine	0.680	0.282	0.592	0.135	0.848	0.714	0.848	0.798	0.584	0.186
pantothenic acid	0.474	0.093	0.468	0.087	0.627	0.243	0.507	0.281	0.941	0.822
phenylalanine	0.569	0.170	0.488	0.110	0.852	0.730	0.553	0.367	0.850	0.650
phosphocholine	0.547	0.043	1.043	0.703	0.813	0.293	1.031	0.928	1.056	0.717
phosphocreatine	0.549	0.087	0.547	0.087	1.081	0.812	0.647	0.367	1.635	0.057
phosphoserine	0.337	0.021	1.392	0.059	0.608	0.119	0.514	0.123	3.238	0.000
proline	0.645	0.203	0.541	0.111	0.848	0.714	0.604	0.404	0.842	0.637
glucose	206.994	0.008	0.632	0.160	1.013	0.971	0.578	0.367	0.870	0.650
SAM	0.737	0.087	0.693	0.052	0.646	0.146	0.972	0.933	0.684	0.085
serine	0.747	0.208	0.635	0.105	0.894	0.730	0.559	0.247	1.018	0.923
taurine	0.626	0.006	0.815	0.022	0.998	0.971	0.952	0.499	0.866	0.144
threonine	0.796	0.228	0.666	0.104	1.070	0.784	0.839	0.575	1.036	0.822
tryptophan	0.606	0.197	0.479	0.110	0.906	0.825	0.584	0.404	0.889	0.739
tyrosine	0.628	0.195	0.495	0.105	0.937	0.889	0.561	0.367	0.835	0.633
UDP	0.564	0.018	0.614	0.019	0.612	0.034	0.868	0.742	0.722	0.063
UMP	0.665	0.040	0.581	0.022	0.787	0.192	0.865	0.627	0.633	0.056
UTP	0.882	0.292	0.794	0.114	0.728	0.243	1.055	0.928	0.779	0.200
valine	1.267	0.058	0.948	0.573	1.684	0.021	1.342	0.166	0.744	0.085
3-phosphoglycerate	1.398	0.043	1.211	0.285	0.907	0.507	1.003	0.968	1.218	0.048
5-amino-levulinic acid	0.700	0.292	0.900	0.573	1.100	0.750	0.900	0.736	1.600	0.085
acetyl-CoA	2.750	0.081	1.125	0.767	1.125	0.911	0.500	0.569	2.500	0.444
adenosine diphosphoribose	1.079	0.291	1.137	0.124	1.005	0.971	1.058	0.712	1.163	0.325

alpha-ketoglutarate	0.571	0.025	0.829	0.105	0.657	0.063	0.629	0.041	0.857	0.151
aminobutyric acid	0.515	0.000	0.672	0.001	0.722	0.001	0.974	0.622	0.932	0.085
citrate	0.727	0.111	0.573	0.051	0.849	0.491	0.800	0.408	0.796	0.212
FAD	0.903	0.282	0.892	0.105	0.978	0.812	1.022	0.822	0.849	0.094
fructose-1,6-bisphosphate	1.812	0.508	0.502	0.041	0.860	0.706	0.976	0.957	1.348	0.700
fumarate	0.700	0.058	2.350	0.008	1.250	0.192	1.325	0.069	1.525	0.014
glucose-6-phosphate	0.808	0.102	0.697	0.022	0.529	0.010	0.912	0.622	0.516	0.010
GDP	0.963	0.663	0.818	0.099	1.159	0.295	0.992	0.957	1.073	0.562
SAH	0.821	0.021	0.661	0.001	1.173	0.192	0.912	0.555	1.073	0.160
glyceraldehyde-3-phosphate	0.909	0.576	1.091	0.425	0.818	0.691	0.818	0.494	1.364	0.758
glycerol-phosphate	0.870	0.043	0.786	0.011	1.908	0.192	0.702	0.035	1.405	0.343
GMP	0.773	0.037	0.605	0.002	1.724	0.202	0.773	0.266	1.357	0.337
lactate	1.036	0.808	0.891	0.425	0.927	0.784	0.964	0.928	1.400	0.083
malate	0.696	0.028	2.224	0.009	1.184	0.192	1.264	0.014	1.440	0.012
N-acetyl aspartate	1.083	0.878	1.083	0.425	1.333	NA	1.333	NA	1.917	0.085
phosphoenolpyruvate	1.419	0.066	1.503	0.025	0.987	0.971	0.858	0.656	1.297	0.085
porphobilinogen	0.800	0.416	1.000	1.000	1.067	0.812	1.067	0.627	0.933	0.650
pyruvate	0.808	0.292	1.268	0.160	1.019	0.949	1.163	0.586	1.686	0.012
xylulose-5-phosphate	0.735	0.171	0.723	0.133	3.193	0.212	0.651	0.324	2.940	0.200
sedoheptulose-7-phosphate	0.300	0.018	0.858	0.363	1.388	0.621	1.014	0.957	1.241	0.396
succinate	0.829	0.027	0.857	0.045	26.800	0.016	0.943	0.627	1.200	0.085
UDP-Glc	0.919	0.018	0.939	0.011	0.909	0.034	1.082	0.024	1.046	0.085
UDP-GlcNAc	0.594	0.001	0.923	0.022	0.988	0.730	1.174	0.004	1.114	0.056
uridine diphosphoglucuronic acid	1.003	0.972	1.148	0.160	1.106	0.703	1.039	0.637	0.875	0.121

SUPPLEMENTARY FIGURES

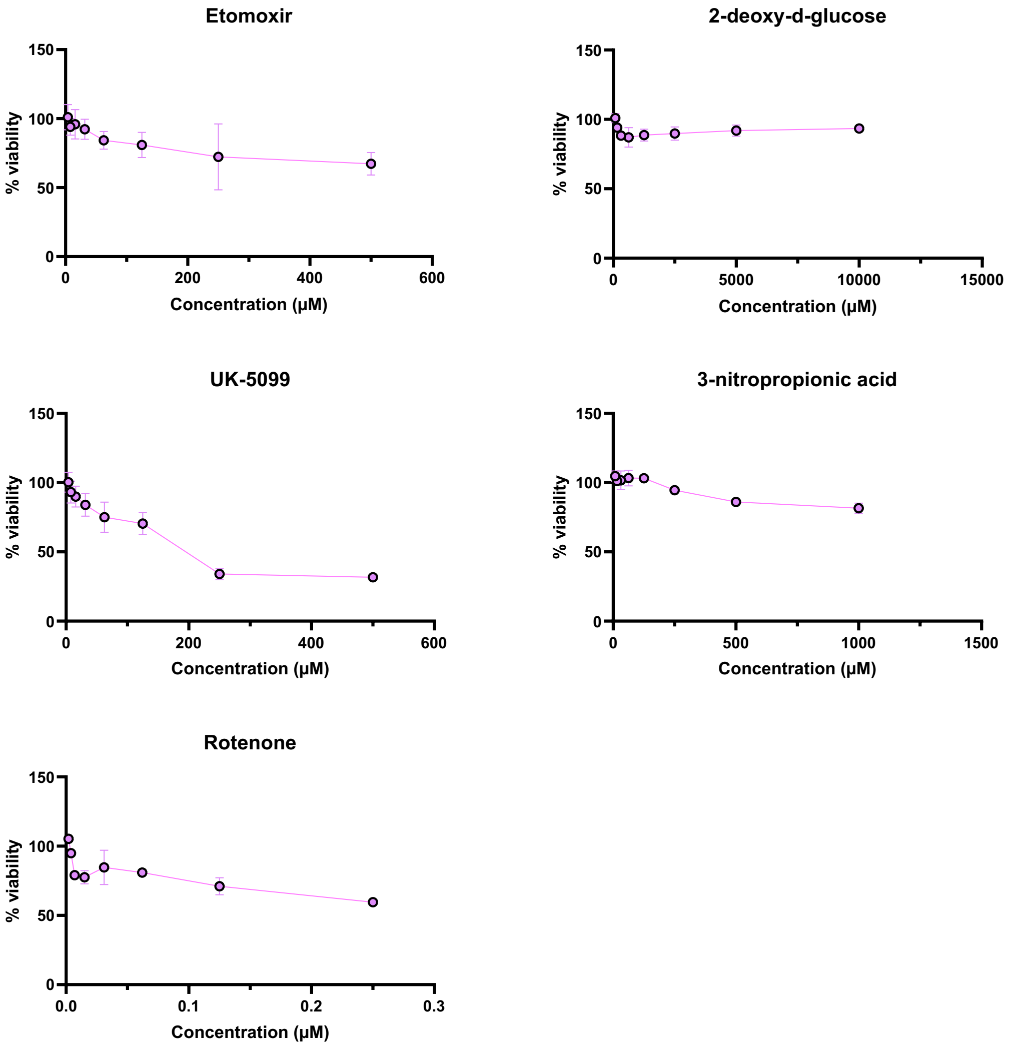

Fig. S1 Viability of HepaRG cells exposed for 24 h with etomoxir, 2-deoxy-D-glucose, UK-5099, 3-nitropropionic acid, and rotenone measured by the resazurin assay. Etomoxir (3.9-500 µM); 2-deoxy-D-glucose (78.1-10’000 µM); UK-5099 (3.9-500 µM); 3-nitropropionic acid (7.81-1000 µM); rotenone (0.002-0.250 µM). N=4; mean±SD.

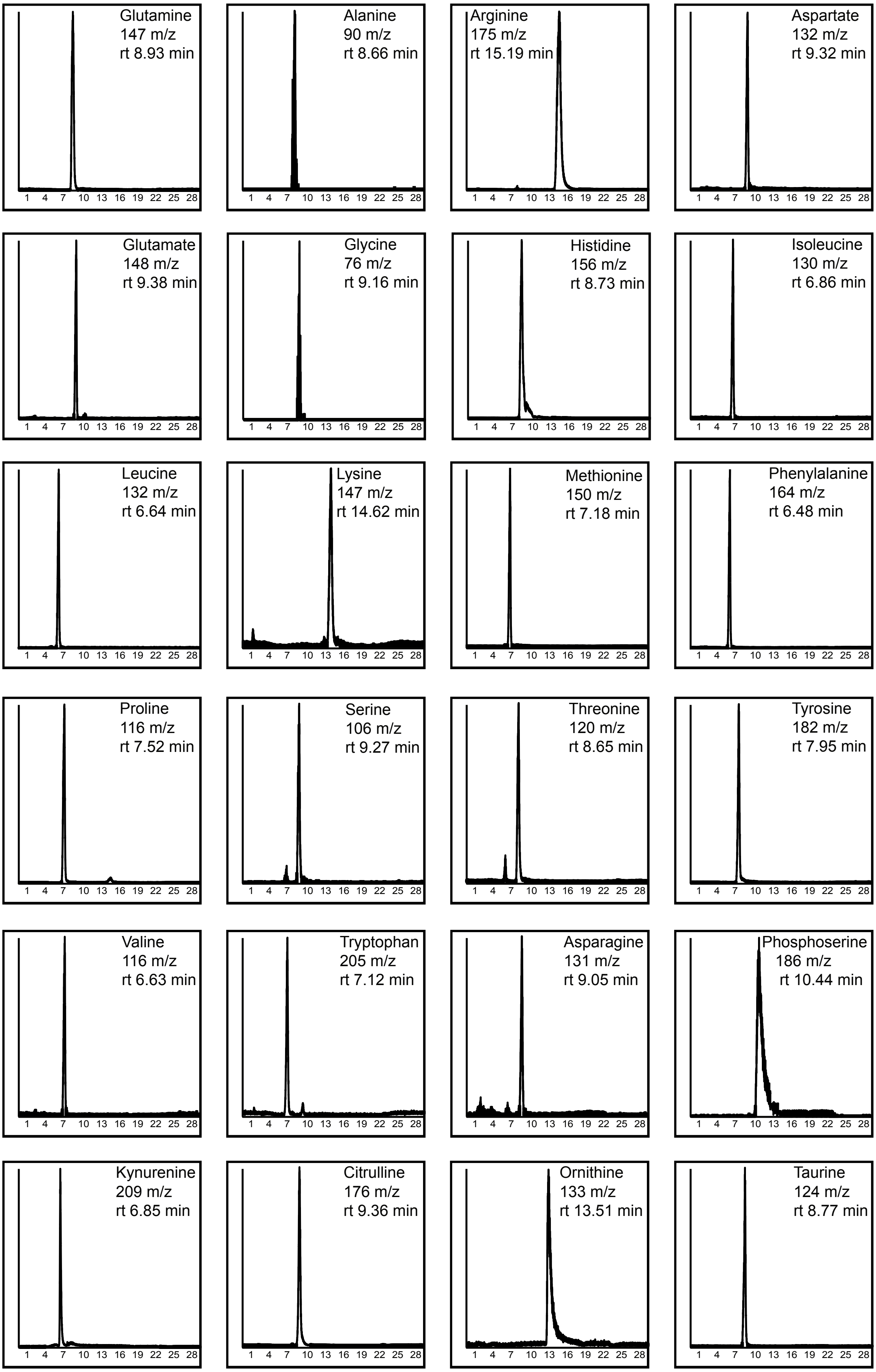
Glutamine
147 m/z
rt 8.93 min
Alanine
90 m/z
rt 8.66 min
Arginine
175 m/z
rt 15.19 min
Aspartate
132 m/z
rt 9.32 min
Glutamate
148 m/z
rt 9.38 min
Glycine
76 m/z
rt 9.16 min
Histidine
156 m/z
rt 8.73 min
Isoleucine
130 m/z
rt 6.86 min
Leucine
132 m/z
rt 6.64 min
Lysine
147 m/z
rt 14.62 min
Methionine
150 m/z
rt 7.18 min
Phenylalanine
164 m/z
rt 6.48 min
Proline
116 m/z
rt 7.52 min
Serine
106 m/z
rt 9.27 min
Threonine
120 m/z
rt 8.65 min
Tyrosine
182 m/z
rt 7.95 min
Valine
116 m/z
rt 6.63 min
Tryptophan
205 m/z
rt 7.12 min
Asparagine
131 m/z
rt 9.05 min
Phosphoserine
186 m/z
rt 10.44 min
Kynurenine
209 m/z
rt 6.85 min
Citrulline
176 m/z
rt 9.36 min
Ornithine
133 m/z
rt 13.51 min
Taurine
124 m/z
rt 8.77 min
1 4 7 10 13 16 19 22 25 28

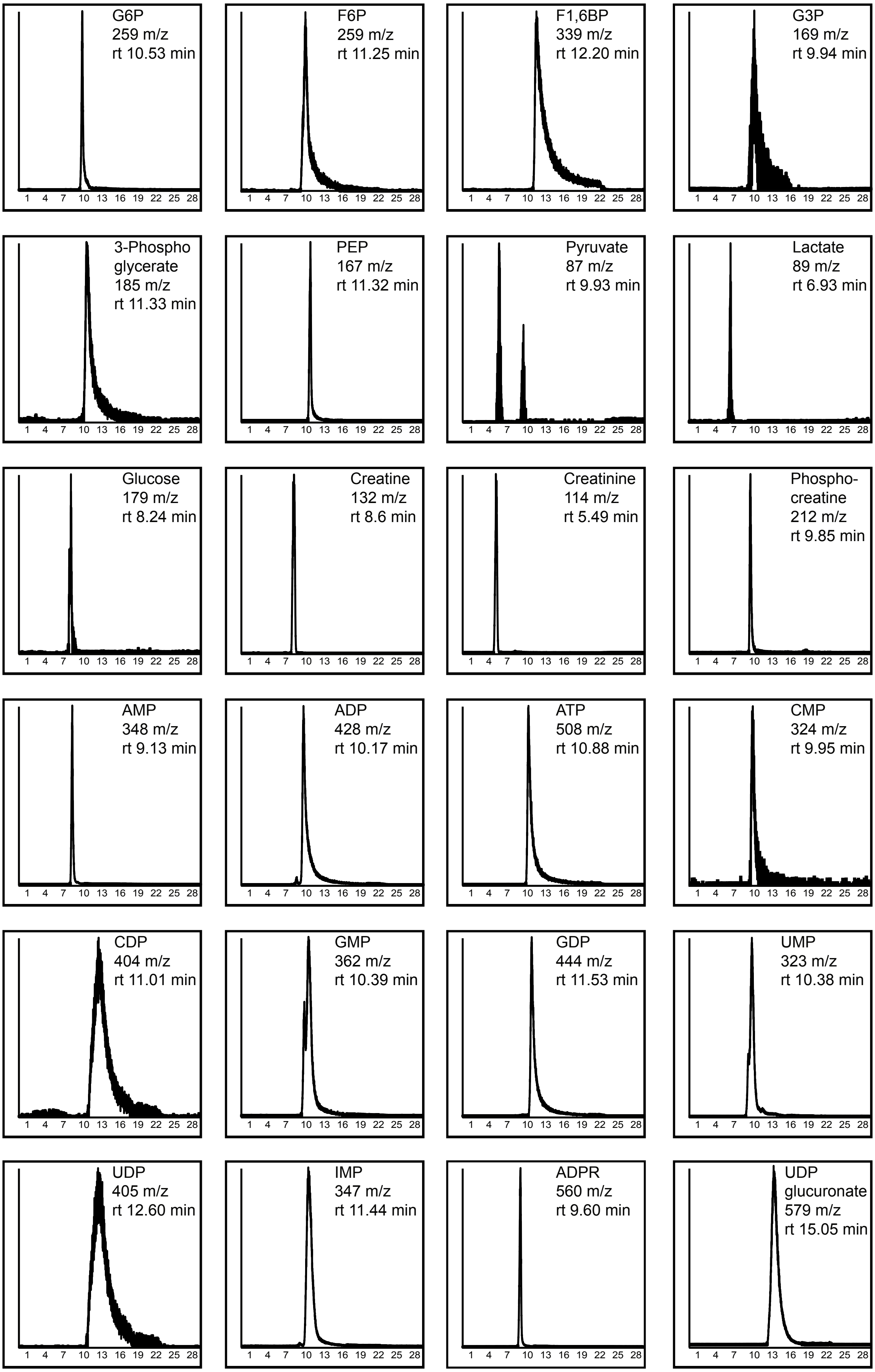

G6P
259 m/z
rt 10.53 min
F6P
259 m/z
rt 11.25 min
F1,6BP
339 m/z
rt 12.20 min
G3P
169 m/z
rt 9.94 min
3-Phospho glycerate
185 m/z
rt 11.33 min
PEP
167 m/z
rt 11.32 min
Pyruvate
87 m/z
rt 9.93 min
Lactate
89 m/z
rt 6.93 min
Glucose
179 m/z
rt 8.24 min
Creatine
132 m/z
rt 8.6 min
Creatinine
114 m/z
rt 5.49 min
Phospho-creatine
212 m/z
rt 9.85 min
AMP
348 m/z
rt 9.13 min
ADP
428 m/z
rt 10.17 min
ATP
508 m/z
rt 10.88 min
CMP
324 m/z
rt 9.95 min
CDP
404 m/z
rt 11.01 min
GMP
362 m/z
rt 10.39 min
GDP
444 m/z
rt 11.53 min
UMP
323 m/z
rt 10.38 min
UDP
405 m/z
rt 12.60 min
IMP
347 m/z
rt 11.44 min
ADPR
560 m/z
rt 9.60 min
UDP glucuronate
579 m/z
rt 15.05 min
1 4 7 10 13 16 19 22 25 28

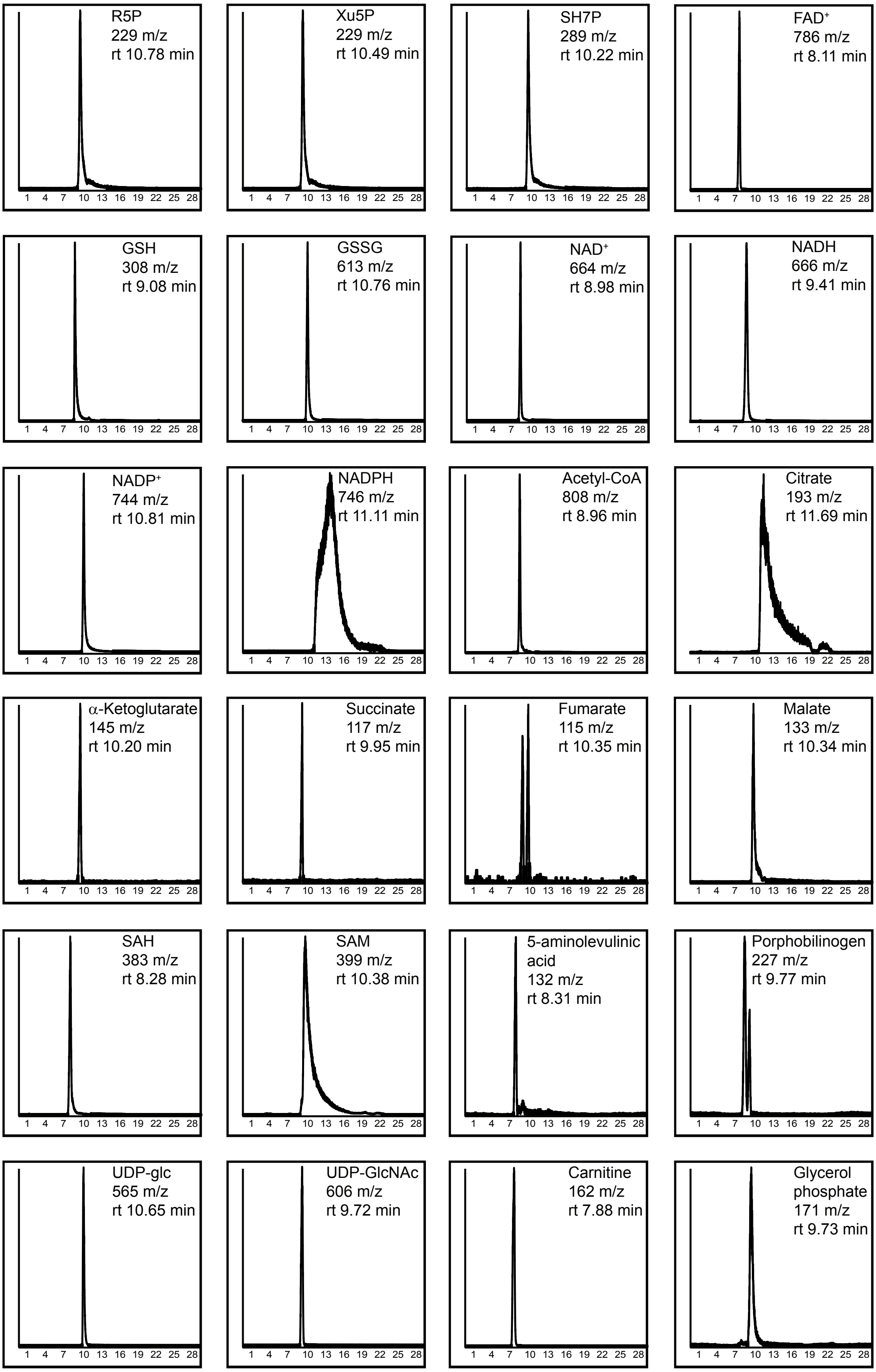
R5P
229 m/z
rt 10.78 min
Xu5P
229 m/z
rt 10.49 min
SH7P
289 m/z
rt 10.22 min
FAD⁺
786 m/z
rt 8.11 min
GSH
308 m/z
rt 9.08 min
GSSG
613 m/z
rt 10.76 min
NAD⁺
664 m/z
rt 8.98 min
NADH
666 m/z
rt 9.41 min
NADP⁺
744 m/z
rt 10.81 min
NADPH
746 m/z
rt 11.11 min
Acetyl-CoA
808 m/z
rt 8.96 min
Citrate
193 m/z
rt 11.69 min
α-Ketoglutarate
145 m/z
rt 10.20 min
Succinate
117 m/z
rt 9.95 min
Fumarate
115 m/z
rt 10.35 min
Malate
133 m/z
rt 10.34 min
SAH
383 m/z
rt 8.28 min
SAM
399 m/z
rt 10.38 min
5-aminolevulinic acid
132 m/z
rt 8.31 min
Porphobilinogen
227 m/z
rt 9.77 min
UDP-glc
565 m/z
rt 10.65 min
UDP-GlcNAc
606 m/z
rt 9.72 min
Carnitine
162 m/z
rt 7.88 min
Glycerol phosphate
171 m/z
rt 9.73 min
1 4 7 10 13 16 19 22 25 28

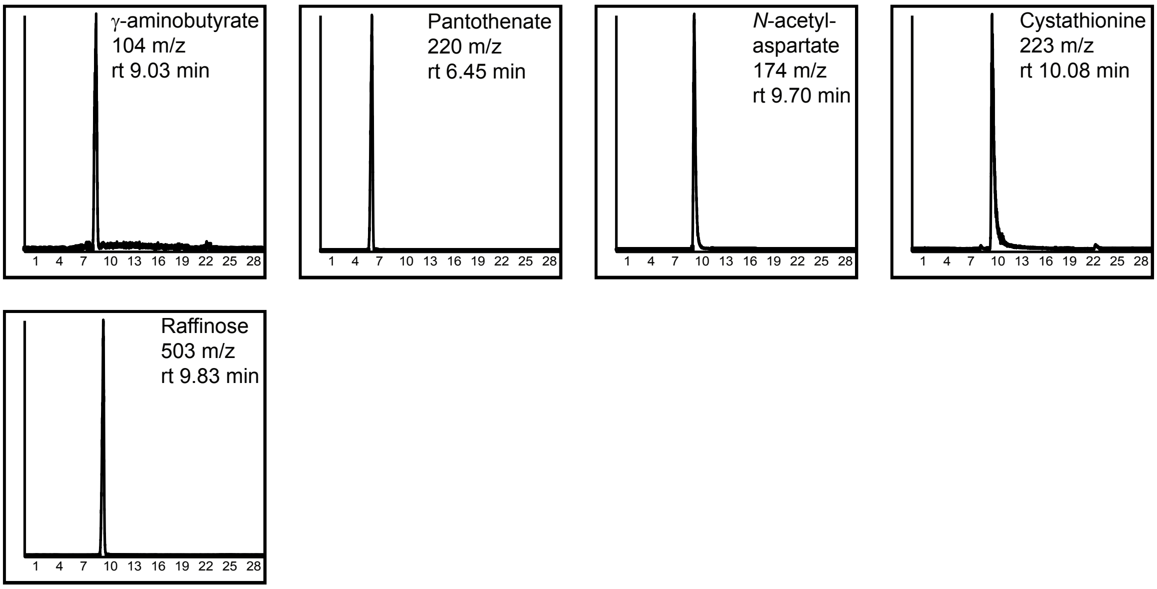

Fig. S2 Chromatograms of 77 metabolites of the central carbon metabolism. Metabolites were either measured by negative or positive ionization mode by LC-MS.

Names of metabolites can be consulted in Table 1.

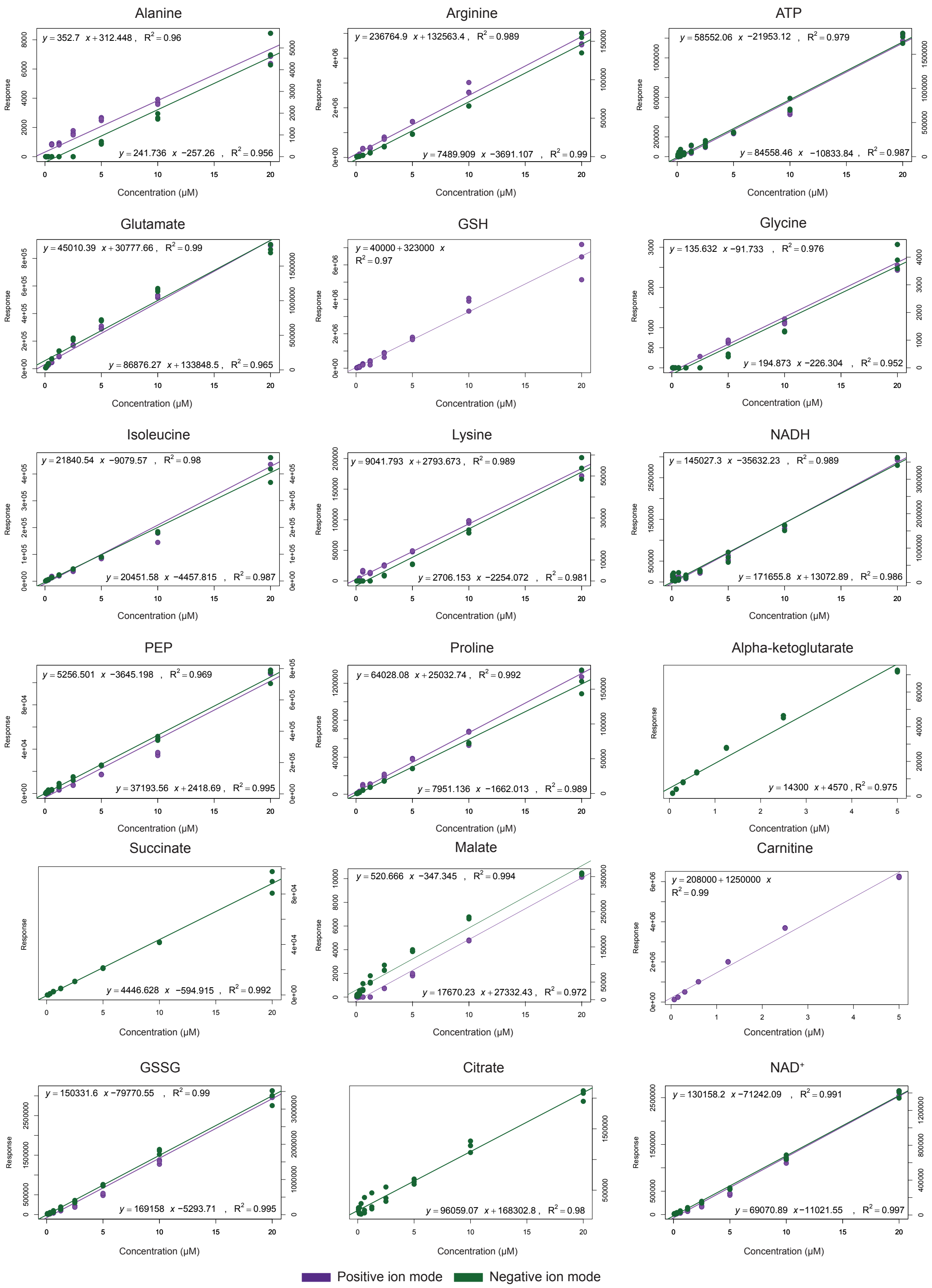
Alanine
y = 352.7 x + 312.448 , R2 = 0.96
y = 241.736 x −257.26 , R2 = 0.956
Arginine
y = 236764.9 x + 132563.4 , R2 = 0.989
y = 7489.909 x −3691.107 , R2 = 0.99
ATP
y = 58552.06 x −21953.12 , R2 = 0.979
y = 84558.46 x −10833.84 , R2 = 0.987
Glutamate
y = 45010.39 x + 30777.66 , R2 = 0.99
y = 86876.27 x + 133848.5 , R2 = 0.965
GSH
y = 40000 + 323000 x
R2 = 0.97
Glycine
y = 135.632 x −91.733 , R2 = 0.976
y = 194.873 x −226.304 , R2 = 0.952
Isoleucine
y = 21840.54 x −9079.57 , R2 = 0.98
y = 20451.58 x −4457.815 , R2 = 0.987
Lysine
y = 9041.793 x + 2793.673 , R2 = 0.989
y = 2706.153 x −2254.072 , R2 = 0.981
NADH
y = 145027.3 x −35632.23 , R2 = 0.989
y = 171655.8 x + 13072.89 , R2 = 0.986
PEP
y = 5256.501 x −3645.198 , R2 = 0.969
y = 37193.56 x + 2418.69 , R2 = 0.995
Proline
y = 64028.08 x + 25032.74 , R2 = 0.992
y = 7951.136 x −1662.013 , R2 = 0.989
Alpha-ketoglutarate
y = 14300 x + 4570 , R2 = 0.975
Succinate
y = 4446.628 x −594.915 , R2 = 0.992
Malate
y = 520.666 x −347.345 , R2 = 0.994
y = 17670.23 x + 27332.43 , R2 = 0.972
Carnitine
y = 208000 + 1250000 x
R2 = 0.99
GSSG
y = 150331.6 x −79770.55 , R2 = 0.99
y = 169158 x −5293.71 , R2 = 0.995
Citrate
y = 96059.07 x + 168302.8 , R2 = 0.98
NAD+
y = 130158.2 x −71242.09 , R2 = 0.991
y = 69070.89 x −11021.55 , R2 = 0.997
Response
Concentration (μM)
Positive ion mode
Negative ion mode

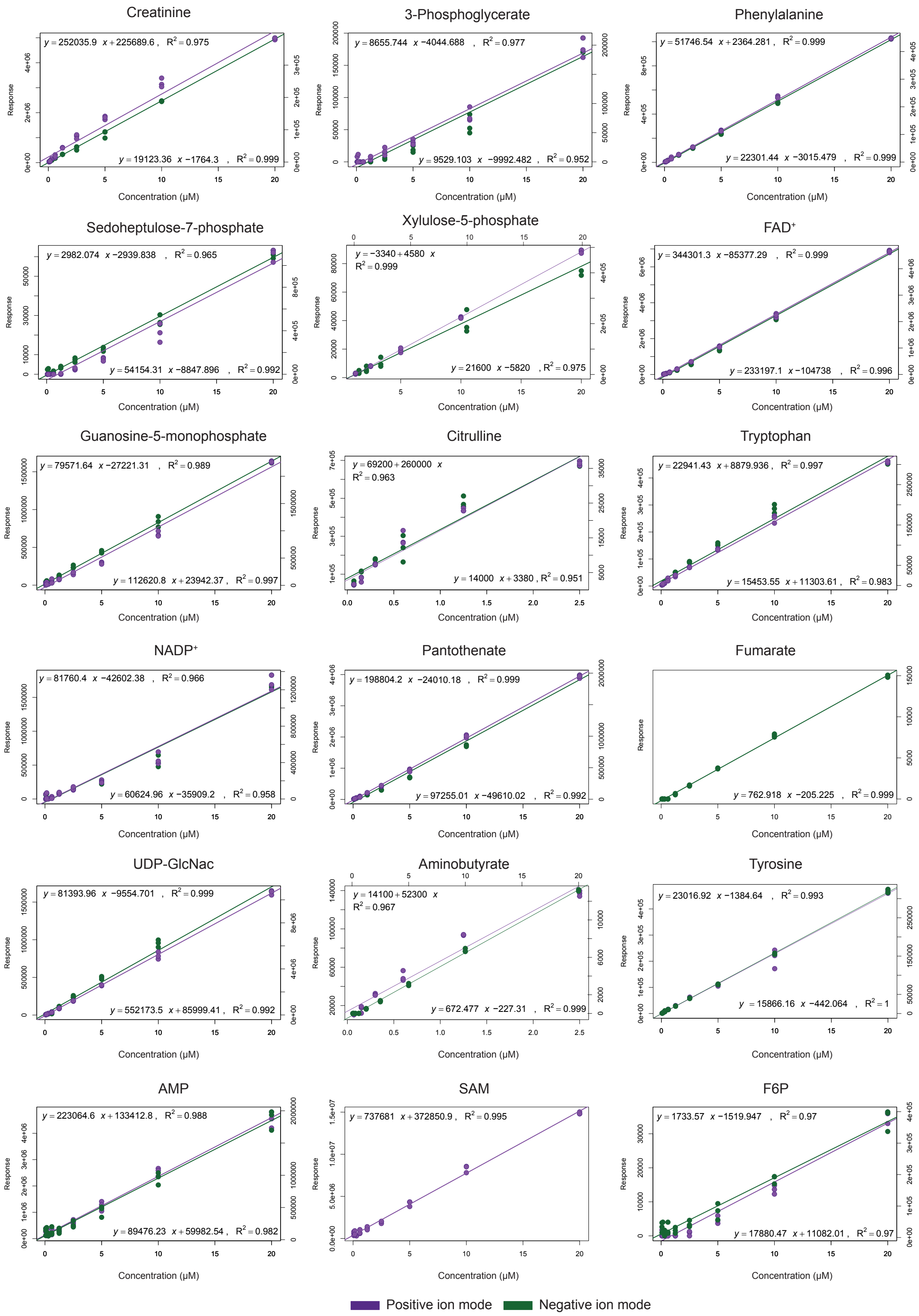
Creatinine
y = 252035.9 x + 225689.6 , R2 = 0.975
y = 19123.36 x −1764.3 , R2 = 0.999
3-Phosphoglycerate
y = 8655.744 x −4044.688 , R2 = 0.977
y = 9529.103 x −9992.482 , R2 = 0.952
Phenylalanine
y = 51746.54 x + 2364.281 , R2 = 0.999
y = 22301.44 x −3015.479 , R2 = 0.999
Sedoheptulose-7-phosphate
y = 2982.074 x −2939.838 , R2 = 0.965
y = 54154.31 x −8847.896 , R2 = 0.992
Xylulose-5-phosphate
y = −3340 + 4580 x
R2 = 0.999
y = 21600 x −5820 , R2 = 0.975
FAD+
y = 344301.3 x −85377.29 , R2 = 0.999
y = 233197.1 x −104738 , R2 = 0.996
Guanosine-5-monophosphate
y = 79571.64 x −27221.31 , R2 = 0.989
y = 112620.8 x + 23942.37 , R2 = 0.997
Citrulline
y = 69200 + 260000 x
R2 = 0.963
y = 14000 x + 3380 , R2 = 0.951
Tryptophan
y = 22941.43 x + 8879.936 , R2 = 0.997
y = 15453.55 x + 11303.61 , R2 = 0.983
NADP+
y = 81760.4 x −42602.38 , R2 = 0.966
y = 60624.96 x −35909.2 , R2 = 0.958
Pantothenate
y = 198804.2 x −24010.18 , R2 = 0.999
y = 97255.01 x −49610.02 , R2 = 0.992
Fumarate
y = 762.918 x −205.225 , R2 = 0.999
UDP-GlcNac
y = 81393.96 x −9554.701 , R2 = 0.999
y = 552173.5 x + 85999.41 , R2 = 0.992
Aminobutyrate
y = 14100 + 52300 x
R2 = 0.967
y = 672.477 x −227.31 , R2 = 0.999
Tyrosine
y = 23016.92 x −1384.64 , R2 = 0.993
y = 15866.16 x −442.064 , R2 = 1
AMP
y = 223064.6 x + 133412.8 , R2 = 0.988
y = 89476.23 x + 59982.54 , R2 = 0.982
SAM
y = 737681 x + 372850.9 , R2 = 0.995
F6P
y = 1733.57 x −1519.947 , R2 = 0.97
y = 17880.47 x + 11082.01 , R2 = 0.97
Response
Concentration (µM)
Positive ion mode
Negative ion mode

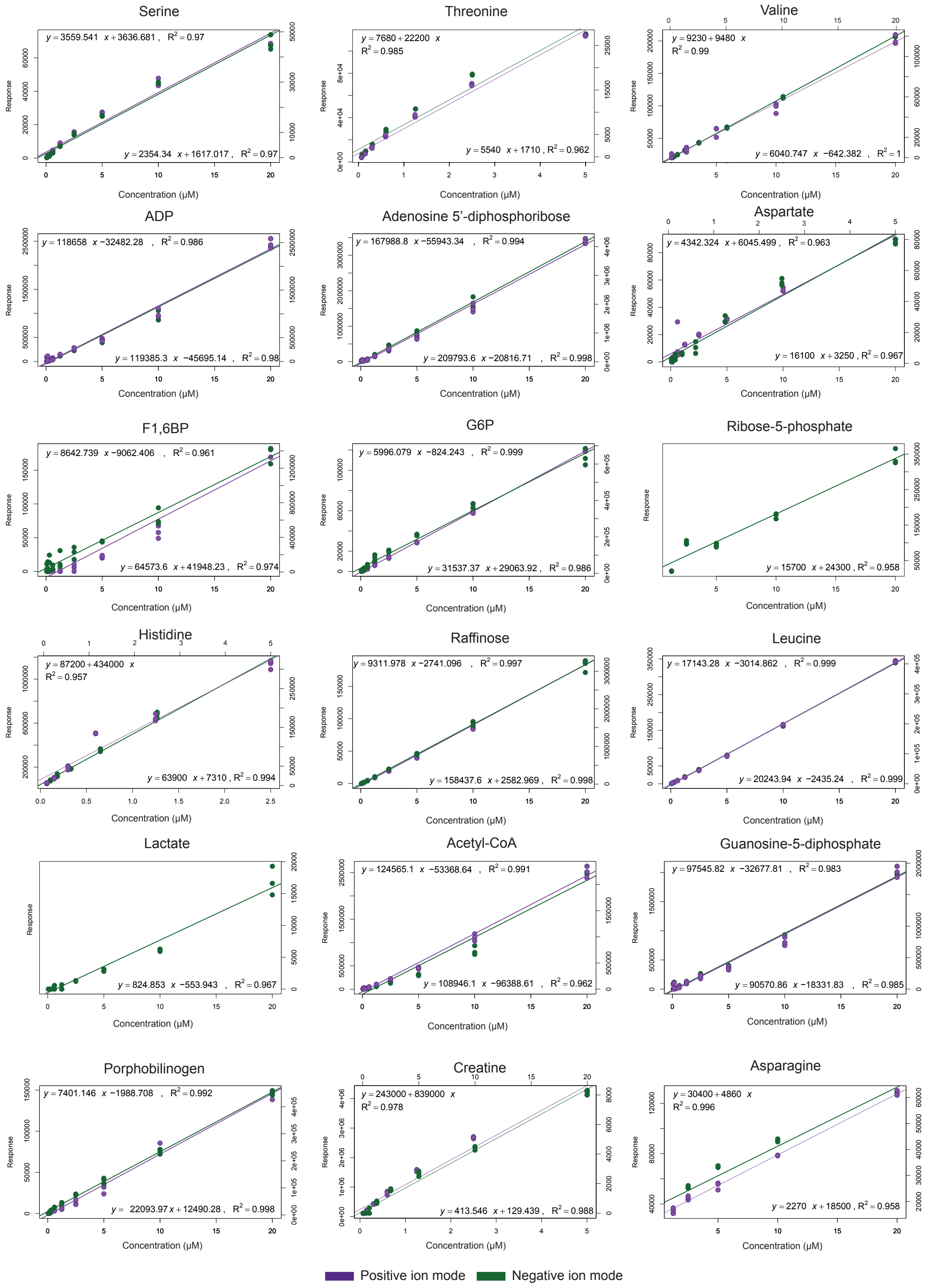

Serine
y = 3559.541 x + 3636.681 , R^2 = 0.97
y = 2354.34 x + 1617.017 , R^2 = 0.97
Threonine
y = 7680 + 22200 x
R^2 = 0.985
y = 5540 x + 1710 , R^2 = 0.962
Valine
y = 9230 + 9480 x
R^2 = 0.99
y = 6040.747 x −642.382 , R^2 = 1
ADP
y = 118658 x −32482.28 , R^2 = 0.986
y = 119385.3 x −45695.14 , R^2 = 0.98
Adenosine 5'-diphosphoribose
y = 167988.8 x −55943.34 , R^2 = 0.994
y = 209793.6 x −20816.71 , R^2 = 0.998
Aspartate
y = 4342.324 x + 6045.499 , R^2 = 0.963
y = 16100 x + 3250 , R^2 = 0.967
F1,6BP
y = 8642.739 x −9062.406 , R^2 = 0.961
y = 64573.6 x + 41948.23 , R^2 = 0.974
G6P
y = 5996.079 x −824.243 , R^2 = 0.999
y = 31537.37 x + 29063.92 , R^2 = 0.986
Ribose-5-phosphate
y = 15700 x + 24300 , R^2 = 0.958
Histidine
y = 87200 + 434000 x
R^2 = 0.957
y = 63900 x + 7310 , R^2 = 0.994
Raffinose
y = 9311.978 x −2741.096 , R^2 = 0.997
y = 158437.6 x + 2582.969 , R^2 = 0.998
Leucine
y = 17143.28 x −3014.862 , R^2 = 0.999
y = 20243.94 x −2435.24 , R^2 = 0.999
Lactate
y = 824.853 x −553.943 , R^2 = 0.967
Acetyl-CoA
y = 124565.1 x −53368.64 , R^2 = 0.991
y = 108946.1 x −96388.61 , R^2 = 0.962
Guanosine-5-diphosphate
y = 97545.82 x −32677.81 , R^2 = 0.983
y = 90570.86 x −18331.83 , R^2 = 0.985
Porphobilinogen
y = 7401.146 x −1988.708 , R^2 = 0.992
y = 22093.97 x + 12490.28 , R^2 = 0.998
Creatine
y = 243000 + 839000 x
R^2 = 0.978
y = 413.546 x + 129.439 , R^2 = 0.988
Asparagine
y = 30400 + 4860 x
R^2 = 0.996
y = 2270 x + 18500 , R^2 = 0.958
Response
Concentration (μM)
Positive ion mode
Negative ion mode

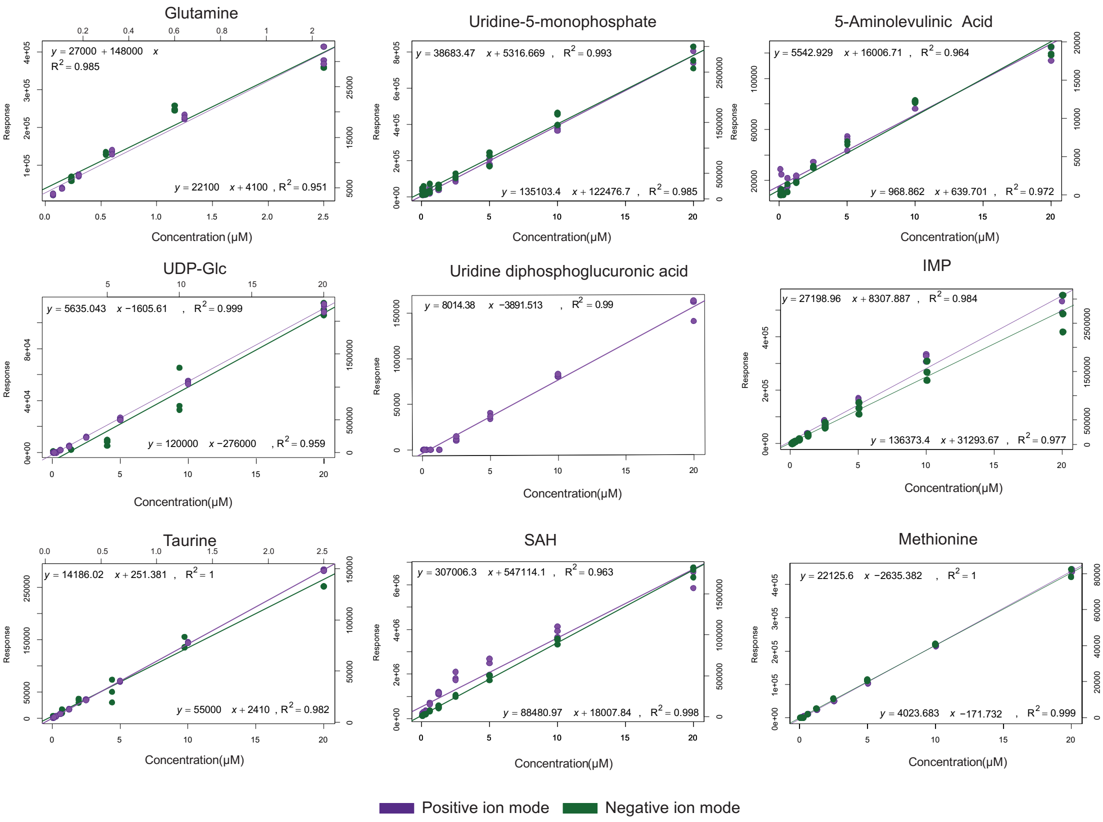

Fig. S3 Standard curves of 63 metabolites of the central carbon metabolism. For each standard curve, 9 concentrations were acquired by LC-HRMS (ESI positive, purple, left y-axis, bottom x-axis; and ESI negative, green, right y-axis, top x-axis when different concentrations are used), relative to the LC-MS instrumental response in function of concentration. Concentrations are between 0.08 and 20 μM, using dilution series steps of 1:2, with displayed linear range, expressed by a linear equation with r^2 > 0.95, at the top (positive) and bottom (negative).

Names of metabolites can be consulted in Table 1.

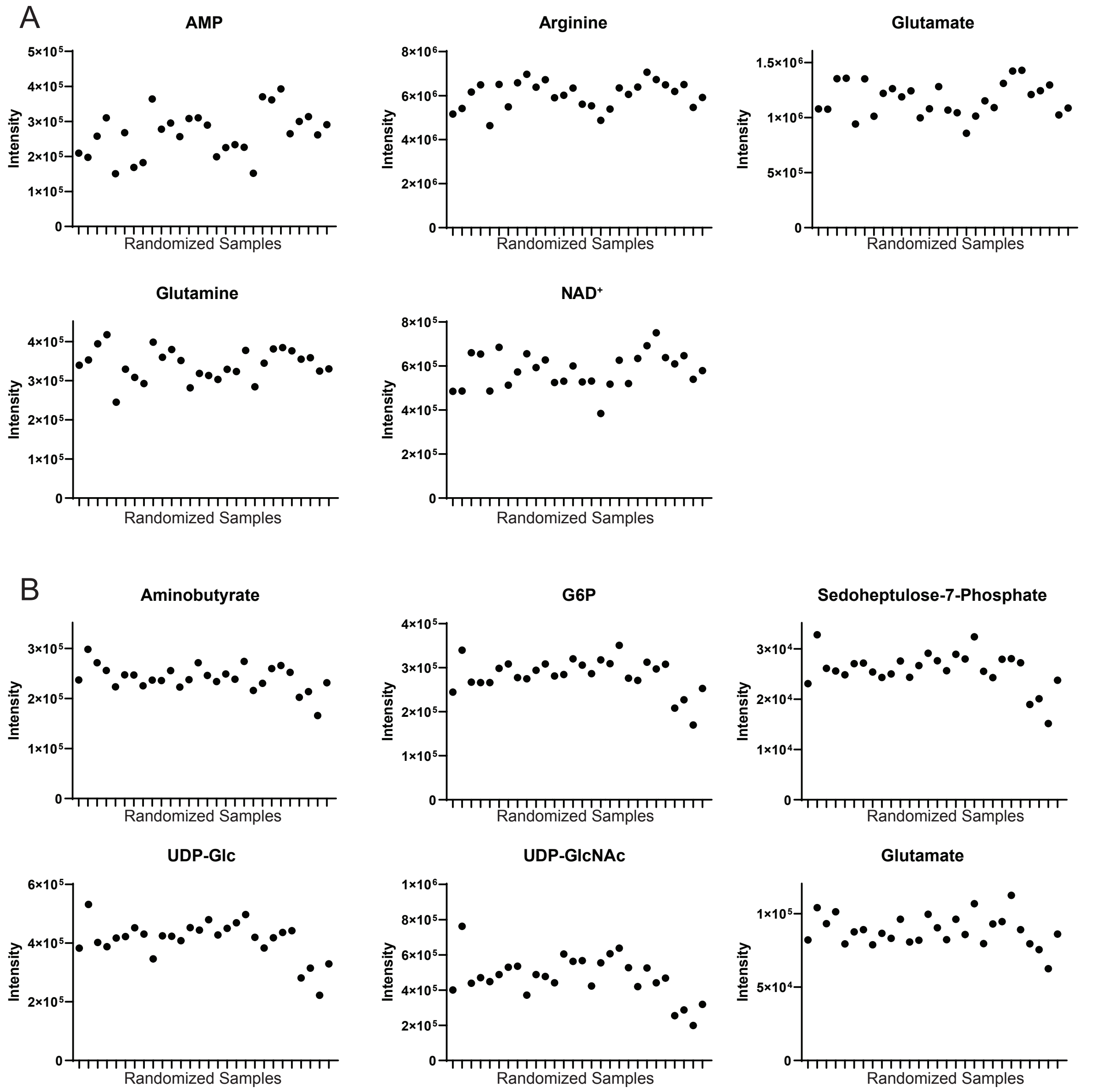

Fig. S4 LC-HR-MS intensities of U-^{13}C-labeled metabolites used as internal standard (IS) across randomized cell extracts and quality control samples (QCs). IS used for **A.** positive and **B.** negative ion mode data normalization

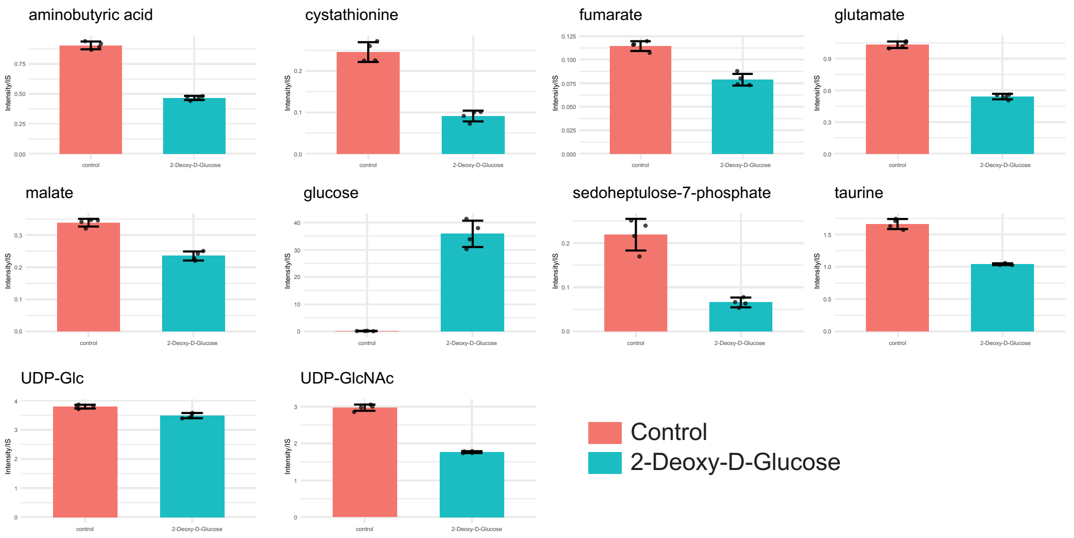

Fig. S5 Top 10 significant metabolites with the lowest adjusted *p*-value by false discovery rate (FDR) in cell extracts challenged by 2-deoxy-D-glucose (turquoise) compared to controls (vehicle, red); N=4, mean ± SD. Only metabolites with *p*-adjusted value <0.05, calculated with a t-test, were considered. Data is represented as LC-MS intensity normalized to IS.

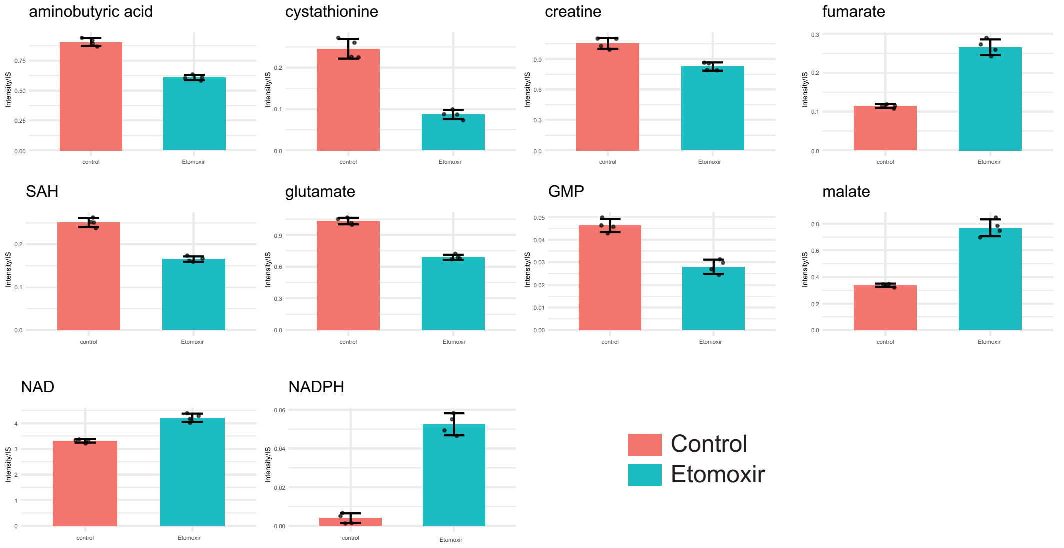

Fig. S6 Top 10 significant metabolites with the lowest adjusted *p*-value by false discovery rate (FDR) in cell extracts challenged by etomoxir (turquoise) compared to controls (vehicle, red); N=4, mean ± SD. Only metabolites with *p*-adjusted value <0.05, calculated with a t-test, were considered. Data is represented as LC-MS intensity normalized to IS.

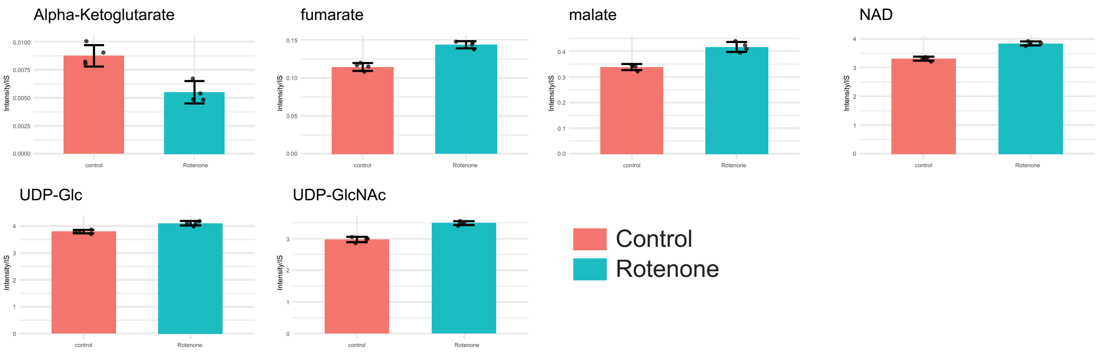

Fig. S7 Top 10 significant metabolites with the lowest adjusted *p*-value by false discovery rate (FDR) in cell extracts challenged by rotenone (turquoise) compared to controls (vehicle, red); N=4, mean ± SD. Only metabolites with *p*-adjusted value <0.05, calculated with a t-test, were considered (except for fumarate FDR-adjusted *p-value*= 0.069). Data is represented as LC-MS intensity normalized to IS.

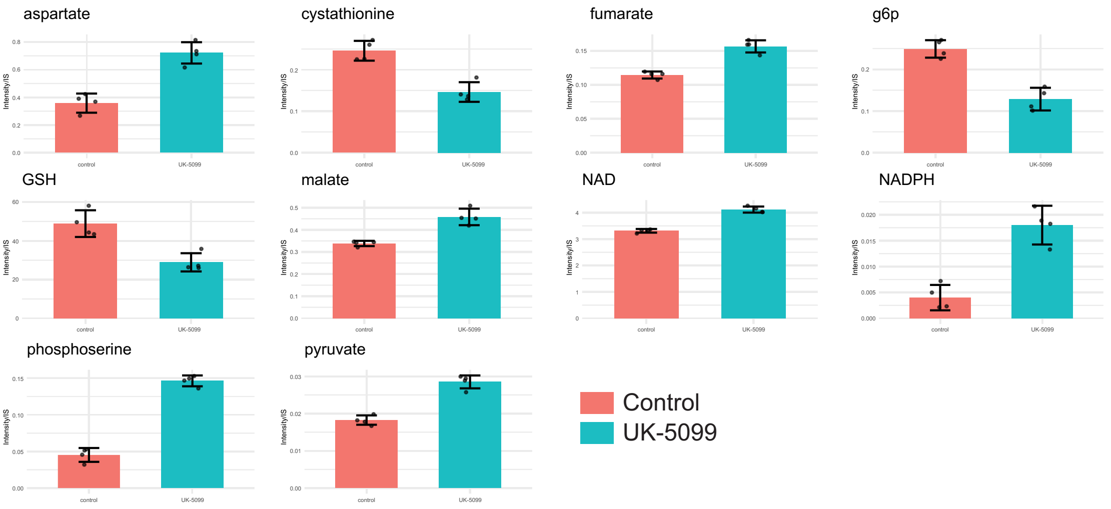

Fig. S8 Top 10 significant metabolites with the lowest adjusted *p*-value by false discovery rate (FDR) in cell extracts challenged by UK-5099 (turquoise) compared to controls (vehicle, red); N=4, mean ± SD. Only metabolites with *p*-adjusted value <0.05, calculated with a t-test, were considered. Data is represented as LC-MS intensity normalized to IS.

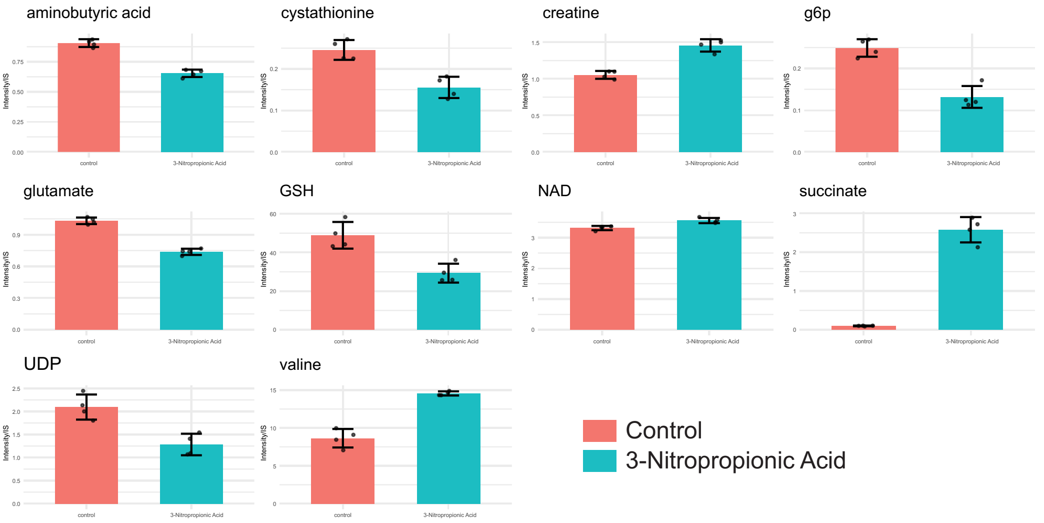

Fig. S9 Top 10 significant metabolites with the lowest adjusted *p*-value by false discovery rate (FDR) in cell extracts challenged by 3-nitropropionic acid (turquoise) compared to controls (vehicle, red); N=4, mean ± SD. Only metabolites with *p*-adjusted value <0.05, calculated with a t-test, were considered. Data is represented as LC-MS intensity normalized to IS.
